# Chaperone-Mediated Autophagy Ablation in Pericytes Reveals New Glioblastoma Prognostic Markers and Efficient Treatment Against Tumor Progression

**DOI:** 10.3389/fcell.2022.797945

**Published:** 2022-03-18

**Authors:** María Luisa Molina, David García-Bernal, María Dolores Salinas, Gonzalo Rubio, Pedro Aparicio, José M. Moraleda, Salvador Martínez, Rut Valdor

**Affiliations:** ^1^ Unit of Autophagy, Immune Response and Tolerance in Pathologic Processes, Biomedical Research Institute of Murcia-Virgen de La Arrixaca (IMIB), Murcia, Spain; ^2^ Instituto de Neurociencias-University Miguel Hernández (UMH-CSIC), San Juan de Alicante, Spain; ^3^ Cell Therapy Unit, IMIB, Murcia, Spain; ^4^ Cell Therapy and Hematopoietic Transplant Group-Medicine Department, University of Murcia (UMU), Murcia, Spain; ^5^ Biochemistry, Molecular Biology, and Immunology Department, UMU, Murcia, Spain; ^6^ Instituto de Neurociencias (UMH-CSIC), CIBER de Salud Mental (CIBERSAM-ISCIII) and Alicante Institute for Health and Biomedical Research (ISABIAL), San Juan de Alicante, Spain

**Keywords:** chaperone-mediated autophagy, pericytes, glioblastoma, tumor, prognosis markers, therapeutical strategy, exofucosylation, immunogenic function

## Abstract

**Background:** The lack of knowledge of the progression mechanisms of glioblastoma (GB), the most aggressive brain tumor, contributes to the absence of successful therapeutic strategies. Our team has recently demonstrated a crucial new role for chaperone-mediated autophagy (CMA) in pericytes (PC)-acquired immunosuppressive function, which prevents anti-tumor immune responses and facilitates GB progression. The possible impact that GB-induced CMA in PC has on other functions that might be useful for future GB prognosis/treatment, has not been explored yet. Thus, we proposed to analyze the contribution of CMA to other GB-induced changes in PC biology and determine if CMA ablation in PC is a key target mechanism for GB treatment.

**Methods:** Studies of RNA-seq and secretome analysis were done in GB-conditioned PC with and without CMA (from knockout mice for LAMP-2A) and compared to control PC. Different therapeutic strategies in a GB mouse model were compared.

**Results:** We found several gene expression pathways enriched in LAMP2A-KO PC and affected by GB-induced CMA in PC that correlate with our previous findings. Phagosome formation, cellular senescence, focal adhesion and the effector function to promote anti-tumor immune responses were the most affected pathways, revealing a transcriptomic profiling of specific target functions useful for future therapies. In addition, several molecules associated with tumor mechanisms and related to tumor immune responses such as gelsolin, periostin, osteopontin, lumican and vitamin D, were identified in the PC secretome dependent on GB-induced CMA. The CMA ablation in PC with GB cells showed an expected immunogenic phenotype able to phagocyte GB cells and a key strategy to develop future therapeutic strategies against GB tumor progression. A novel intravenous therapy using exofucosylated CMA-deficient PC was efficient to make PC reach the tumor niche and facilitate tumor elimination.

**Conclusion:** Our results corroborate previous findings on the impaired immunogenic function of PC with GB-induced CMA, driving to other altered PC functions and the identifications of new target markers related to the tumor immune responses and useful for GB prognosis/therapy. Our work demonstrates CMA ablation in PC as a key target mechanism to develop a successful therapy against GB progression.

## Introduction

Glioblastoma multiforme (GB), is considered by the U.S. National Cancer Institute as the most aggressive form of brain cancer. GB represents 15.4% of all primary brain tumors and about 60–75% of all astrocytomas, and shows rapid growth rate of malignant cells in the organ, with a survival rate of 14–15 months. There is no curative treatment available, and new therapies and prognostic factors are unmet needs for GB (Arvanitis et al., 2020; Sampson et al., 2020).

During GB development, the tumor cells infiltrate and invade the cerebral parenchyma interacting with the cells of the perivascular areas and establishing a functional network (Carmeliet and Jain, 2011; Hjelmeland et al., 2011; Osswald et al., 2015; Valdor et al., 2017; Valdor et al., 2019). PC are perivascular stromal cells with stem cell like-properties that can phagocyte and promote an immune defense in response to brain damage (Peppiatt et al., 2006; Bell et al., 2010; Winkler et al., 2011; Gaceb et al., 2018; Molina et al., 2019). We have previously shown that by direct interaction with GB cells, PC acquire an immunosuppressive function during the progression of GB that contributes to the establishment of immunotolerance and, therefore, to tumor growth. This immune function depends on the aberrant upregulation of GB-induced CMA through cell-cell interactions that causes PC to present an anti-inflammatory phenotype and inactivate the T-cell responses for tumor removal (Valdor et al., 2017; Valdor et al., 2019). Therefore, as CMA is universal in all types of cells, including tumor cells, a better understanding of the biology of these cells that surround the tumor is needed to find target specific markers and establish an effective selective therapy (Molina et al., 2019).

CMA, unlike other types of autophagy, selectively degrades soluble cytosolic proteins containing a specific motif (KFERQ) in their amino acid sequence (Kirchner et al., 2019). This motif is recognized by an HSC70 chaperone, which leads the protein to the lysosomal membrane and promotes its interaction with the LAMP-2A lysosomal receptor and its subsequent degradation by the lysosome. CMA activity is directly dependent on the levels of LAMP-2A at the lysosomal membrane, since the binding of substrate proteins to LAMP-2A is the limiting step of this pathway (Kaushik et al., 2011; Kaushik and Cuervo, 2018). This process is critical to maintain cellular function (Kiffin et al., 2004; Kaushik et al., 2011; Valdor et al., 2014; Kaushik and Cuervo, 2018; Valdor et al., 2019) and its activity is upregulated in most tumors, including GB (Kon et al., 2011; Saha, 2012; Quintavalle et al., 2014; Zhou et al., 2016; Arias and Cuervo, 2020). CMA blockage in cancer cells has anticancer activity, decreasing cell proliferation and tumorigenic/metastatic ability (Kon et al., 2011; Arias and Cuervo, 2020). Transformed cells show up-regulated CMA that results in high tolerance to oxidative stress (Saha, 2012), degradation of negative regulators of cell proliferation (Zhou et al., 2016) and anti-oncogenes (Quintavalle et al., 2014) and maintain the metabolic switch favorable for cancer cell growth (Lv et al., 2011; Saha, 2012; Lu et al., 2014). CMA is also described as a regulator mechanism of the immune function in some types of immune cells and its failure, even in aging, may contribute to alter the anti-tumor response (Valdor and Macian, 2012; Macian, 2019; Molina et al., 2019). From our previous experience, we know that CMA inhibition might affect tumor antigen-specific T cell responses (Valdor et al., 2014) and thus, it is important to consider a GB therapy combined simultaneously with another therapeutic strategies that boost anti-tumor immune responses, allowing the complete removal of the tumor. We had showed evidence that the ability of GB-induced CMA in PC to selectively degrade specific proteins, ablates PC immune function (Valdor et al., 2019). Furthermore, identification of degraded specific proteins and other important functions of PC that modulate tumor progression may constitute promising new target mechanisms to treat this aggressive disease. The tumor cells do not only need CMA to proliferate (Arias and Cuervo, 2020), but CMA also modulates mechanisms in PC of the peritumoral microvasculature for its own benefit and survival (Molina et al., 2019; Valdor et al., 2019).

In this work we hypothesized that CMA, as a protein selective degradation mechanism, might represent a novel target for the local control of both PC-tumor cell interaction, and PC modulation of the anti-tumor immune response that facilitates tumor growth. Accordingly, to determine if CMA ablation in PC would still allow these cells to reach the tumoral niche and eliminate tumor cells, and therefore, be an useful approach for the settlement of future therapies, we analyzed the tumor growth of a previously described xenograft mouse model of GB (Valdor et al., 2017; Valdor et al., 2019) after intracranial grafts of PC, and also, after intravenous injection of exofucosylated PC. Exofucosylation has been previously described as a glycan engineering tool to transiently convert membrane native CD44 into the sialofucosylated glycoform known as hematopoietic cell E-/L-selectin ligand (HCELL), the most potent E-selectin receptor (Sackstein et al., 2008; Abdi et al., 2015). As brain tumor endothelial cells in GB has been shown to overexpress E-selectin (Tabatabai et al., 2008), we sought to investigate if PC migration to GB tumoral niche after intravenous administration could be licensed through exofucosylation to enforce HCELL expression in PC, and if this strategy could impact PC therapeutic effects on GB progression.

Furthermore, the identification of new markers related to GB-induced CMA in PC might be essential for the diagnosis and prognosis of the GB cancer. Indeed, interference with the immunosuppressive function of PC through CMA blocking besides reducing tumor growth, may represent a novel target for the development of new therapies against GB and other tumors arising in microvascularized tissues containing PC.

The bioinformatic analysis validations of RNAseq and proteomics studies allowed us to corroborate the relevance of a recovered immunogenic function against tumor cells in CMA-deficient PC. Moreover, we have identified new proteins in the secretome of PC, as potential prognostic markers of GB progression that need to be validated in future studies in humans. In addition, our data revealed new disrupted PC functions such as phagocytosis, and provided better knowledge of other possible causes of GB-induced dysregulation of CMA in PC. Notwithstanding, the basic mechanisms underlying the aberrant upregulation of GB-induced CMA in PC and the identification of CMA protein substrates that trigger changes in PC function and promote PC-GB interactions to facilitate tumor survival, need to be elucidated in future studies. Our data indicate that CMA-deficient PC have potential to reach the brain tumor areas, prevent PC-GB interactions, promote tumor cell elimination by the anti-tumor immune response and therefore, might help to develop new and effective therapeutical strategies in future preclinical studies in GB.

## Material and Methods

### Mice

Males and females mice of eight to 12 weeks old WT C57BL/6 and C57Bl/6-Tg (ACTB-EGFP)1Osb/J (Charles River Laboratories) were maintained in pathogen-free conditions in the animal facilities of the University of Murcia and Biomedical Research Institute of Murcia Virgen de la Arrixaca. All animal procedures were approved and performed according to the guidelines set by the University of Murcia Institutional Animal Care and Use Committee.

### Cell Culture

Primary brain PC (WT PC and GFP-PC) from mice were isolated and co-cultured with GB cells at a ratio 1:1 for 72 h, as described previously (Valdor et al., 2017). PC with impaired CMA (KO PC), were isolated from brains of Lamp2a−/− mice (Schneider et al., 2015). Human GB cell lines U373-MG and U87 were purchased from European Collection for Authenticated Cell Cultures. For secretome analysis, cell culture media obtained from 72 h co-cultures of GB and PC was concentrated using Amicon Ultra centrifugal filters 10k (Millipore) and used 10 times diluted as described previously (Valdor et al., 2019). DiI labeling solution (Invitrogen) and GFP-expressing PC were used for cell tracking as described previously (Valdor et al., 2019).

### RNA Sequencing and Differential Expression Analysis

For RNA-seq, total RNA from WT PC, KO PC and GB, single and co-cultured PC-GB, was extracted with the purification RNA RNeasy Mini Kit following manufacturer instructions and treated with DNase I (Qiagen). Equal amounts of purified total RNA from three to four experiments of each one were pooled in each sample. DNA libraries for small RNAs and mRNAs were processed and sequenced by the CRG core genomics facility (Barcelona, Spain) using a HiSeq-2500 apparatus (Illumina, service provided by Fasteris S.L.) according to the manufacturer’s instructions. For the quality control, read alignment, obtaining metrics for gene expression, please *see* Supplementary Text. Differentially expressed genes (DEGs) between GB conditioned PC (GB-WT PC) and CMA-deficient PC with GB (GB-KO PC) were detected using DESeq2 v1.18.1 package (Love et al., 2014) in R computing platform v3.4.4 (Ihaka and Gentleman, 1996). DEGs were computed using batch correction in the formula design (design = ∼condition + sample_batch). Genes with FDR Adj. *p* < 0.01 were considered significantly differentially expressed. Raw data are publicly available in the European Nucleotide Archive ENA (study ID PRJEB48545).

### Heatmap, Functional Annotation and Pathway Analyses

A heatmap was generated to visualize the expression values of the interest up-regulated genes and another for the visualization of the down-regulated ones in GB-KO PC vs GB-WT PC with FDR <0.01. To generate the heatmap, the heatmap.2 function of the R (R Core Team, 2021) g plots package was used. Network visualization of Gene Ontology enrichment of proteins of the main affected up-regulated or down-regulated pathways was performed by STRING v11.5 functional protein association networks. Major clusters are circled and node size indicates the number of proteins per node.

### *In Vitro* Phagocytosis Assay

A protocol similar to that described in Diaz-Aparicio et al. was followed (Diaz-Aparicio et al., 2016). Briefly, WT PC and KO PC were allowed to rest and settle for at least 48 h before phagocytosis experiments in 24-well plates. GB cell lines were previously labeled with the cell tracker DiI and treated with 60 μM of staurosporine (Cayman Chemical) for 48 h to induce apoptosis. Only the floating dead-cell fraction was collected from the supernatant and added to the PC cultures in a proportion of 1:1. Apoptotic cells were visualized and quantified by trypan blue exclusion. Because cell membrane integrity is still maintained in early induced apoptotic cells, cells not labeled with trypan blue were considered apoptotic. After 2 h, cells were fixed with 4% paraformaldehyde in PBS after washing away with media to discard all apoptotic cells non-trapped by PC. Due to its non-adherent nature, after cell washing, only apoptotic cells that have been trapped by PCs (in different stages of phagocytosis) remain. Remanent apoptotic cells trapped by PC and PC were stained with AlexaFluor 488-labeled Phalloidin (Invitrogen) to detect F-actin cytoskeleton. Images were acquired with a Delta Vision RT (Applied Precision) restoration microscope coupled to a Coolsnap HQ camera (Photometrics), with a 60×/1.42 Plan Apo or 100×/1.40 Uplan Apo objectives. The percentage of PC with phagocytic pouches (Ph capacity) was counted as described previously (Diaz-Aparicio et al., 2020). Morphometric measurements and quantification of cells were performed using ImageJ (NIH, United States) software. Pictures for illustrations and quantitative analysis were uploaded from direct microscopic images and were not manipulated in subsequent steps of figures preparation, except for framing and scaling.

### Real-Time PCR (qPCR)

cDNA was synthesized from total mRNA, and gene expression was analyzed by real-time PCR using SYBR Green in a Step One Plus Thermocycler (Applied Biosystems), as described previously (Valdor et al., 2017). For primer sequence information, please *see* Supplementary Text.

### Secretome Analysis

Differential expression of secreted proteins in WT PC + GB or KO PC + GB was qualitatively analyzed, identifying the proteins related to pro-tumor or anti-tumor mechanisms and according to Biological Processes of Gene Ontology. For previous sample preparation, secretome protein identification and analysis by HPLC/MS system, please *see* Supplementary Text. The default set of threshold was log_2_FoldChange ≥1.25 for up-regulated proteins in KO PC + GB, and log_2_FoldChange ≤ −1.25 for down-regulated proteins in KO PC + GB, following the recommendations previously reported (Pascovici et al., 2016). From this analysis, we focus on the secretion of those molecules of interest for this study. Levels of secretion of those molecules that were associated with the immune response and with the highest level of protein secretion detected, were measured by ELISA.

### ELISA

PC (5 × 10^4^) were co-cultured with GB cells at 1:1 ratio in 96-well plates for 72 h. Mouse gelsolin, periostin (Wuhan Fine Biotech co.), osteopontin (Abclonal), lumican and 25-HO Vitamin D (Arigo Biolaboratories) levels secreted by PC in the media were measured by sandwich ELISA with specific anti-mouse antibodies following the manufacturer’s recommendations.

### PC Exofucosylation

Murine pericytes were modified by enzymatic exofucosylation as previously reported (Garcia-Bernal et al., 2020). Briefly, cells were resuspended at 2 × 10^7^ cells/ml in fucosyltransferase VII (FTVII) reaction buffer composed of Hanks Balanced Salt Solution (HBSS, Gibco) containing 30 μg/ml fucosyltransferase VII (FTVII, R&D Systems), 20 mM HEPES (Thermo Fisher Scientific), 0.1% human serum albumin (Grifols) and 1 mM guanosine 5′-diphospho-β-L-fucose sodium salt (GDP-fucose, Sigma Aldrich), and incubated for 60 min at 37°C and 5% CO_2_. Unmodified controls pericytes were treated only with GDP-fucose (w/o FTVII) in the same conditions as above. Cell viability after exofucosylation was assessed by trypan blue exclusion (usually 95% live cells). Efficacy of exofucosylation was evaluated by analysis of HECA452 antibody (BD Biosciences) staining and calcium-dependent mouse E-selectin/human IgG chimera (R&D Systems) binding by flow cytometry.

### Xenografts and Therapeutical Strategies

Cell pellets (5 × 10^6^ cells) from human GB cells (U87 and U373 GB lines) were grafted into C57BL/6 wild type mice brains. Xenografts were performed as previously described in an immunocompetent mouse model (Valdor et al., 2017). Cell pellets, prepared as hanging drops, were grafted into mice. Xenografts (1 pellet/mouse) were introduced into the right hemisphere through a small craniotomy (2–3 mm from the midline, approximately 1 mm behind the bregma) at 2.5 mm depth, using a stereotactic apparatus and a Pasteur pipette hand-pulled to an internal diameter of 0.38 mm. This produced grafts that integrated into the occipital cortex or the hippocampus. Three weeks post-grafting, mice were treated with different therapeutical strategies to compare to those none treated. Five mice were intracraneally grafted with KO PC (intracranial therapy, IC therapy) through the previous craniotomy as above described for GB xenografts (1.5 × 10^6^ cells); five mice were injected intravenously (0.5 × 10^6^ cells per tail; 200 μl) with exofucosylated WT or KO PC (Intravenous therapy, IV therapy); and five mice were injected intravenously with unmodified or exofucosylated GFP-PC (0.5 × 10^6^ cells per tail; 200 μl). Four weeks after therapies, mice were sacrificed and brains were fixed in 4% buffered formalin (Panreac Quimica). All procedures were repeated three times and analyzed in each experimental line (GB, IC-KO PC, IV-WT PC, IV-KO PC, IV-GFP-PC; IV-Fuco-GFP-PC).

### Immunohistochemistry

Brains were paraffin embedded and processed by the Pathology facility (IMIB Virgen de la Arrixaca) as described previously (Valdor et al., 2019). Three-micrometer thick serial sections were obtained from paraffin embedded samples using an automatic rotary microtome (Thermo Scientific). For colorimetric immunolabeling, sections were incubated overnight at 4°C with mouse anti-human STEM121 (Cellartis), rabbit anti-GFP (Abcam), goat anti-Iba-1 (Abcam) and rat anti-CD68 (Abdserotec) primary antibodies. Sections were finally incubated with the corresponding 3-3′Diaminobencidine (DAB) secondary antibodies (Vector Labs) and hematoxylin counterstained. Positive immunoreaction was identified as a dark-brown precipitated. An automatic digital slide scanner (Pannoramic MIDI II-3DHistech) and Quantitative Pathology & Bioimage Analysis Qupath-0.2.3 software were used for analysis of histological sections, and acquisition of images. Morphometric measurements and quantification of cells were also performed using ImageJ (NIH, United States) software.

### Flow Cytometry Analysis

Mononuclear cells from central draining lymph nodes of mice were isolated and labeled using specific antibodies for murine CD4 (eBioscience), PD-1 (Novus), FoxP3 (eBioscience) and CTLA-4 (eBioscience). Background nonspecific fluorescence was measured using control isotype antibodies. Labeled cells were analyzed by flow cytometry using a FACS Canto II flow cytometer (BD Bioscience) and data analyzed with Kaluza analysis software (Beckman Coulter). All determination were performed in at least *n* = 5 mice per experimental group and three separate experiments.

### Statistical Analysis

Differences between groups were analyzed by one-way ANOVA followed by Tukey-Kramer posttest. Comparisons between data pairs were analyzed using a *t* test. Statistical significance was defined as *p* < 0.05.

## Results

### Transcriptomic Profiling in CMA-Deficient PC in Response to GB Reveals Specific Target Functions Useful for Future Therapy

**FIGURE 1:**
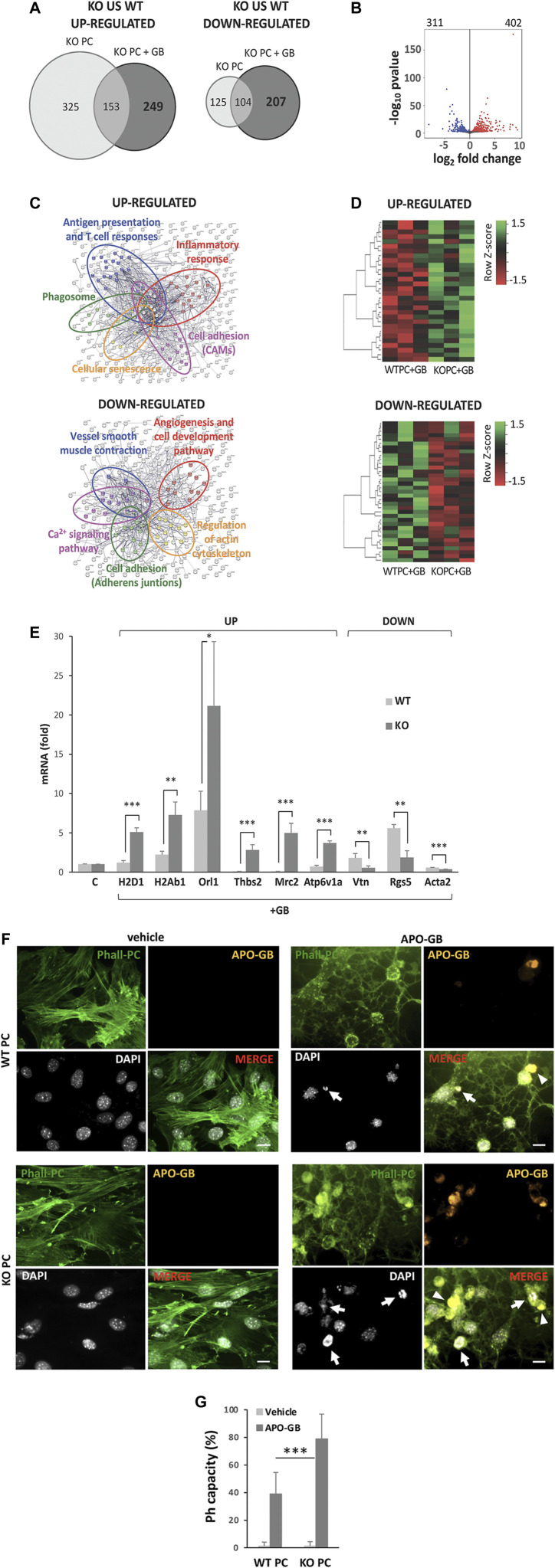
CMA-dependent DEGs and main pathways affected. **(A)** Venn diagram of significantly differential expression of genes (DEGs) “up-regulated” or “down-regulated” in KO PC compared to WT PC in the presence or the absence of GB (FDR<0.01). **(B)** Volcano plot of all significant DEGs in KO PC + GB compared to WT PC+GB. In red are shown the 402 up-regulated genes and in blue, the 311 down-regulated genes. The default set of threshold was FDR<0.01. **(C)** Network visualization of Gene Ontology enrichment of proteins of the main selected affected up-regulated (anti-tumoral; above) or down-regulated (pro-tumoral; below) pathways from CMA-dependent DEGs. Major clusters are circled and node size indicates the number of proteins per node. **(D)** Heatmap of up-regulated CMA-dependent DEGs (above) and down-regulated CMA-dependent DEGs (below) corresponding to the main affected pathways in all the three samples of each condition (WT+GB 1, WT+GB 2, WT+GB 3, KO+GB 1, KO+GB 2, KO+GB 3). Red boxes represent down-regulated genes, and green boxes represent up-regulated genes. The value of expression intensity is based on the gene expression level analysis. All previous data were obtained from three RNA pools for each experimental line, of at least, five independent experiments using U87 GB line. **(E)** Validation and quantification of the mRNA expression by qPCR, of some of the most up-regulated and down-regulated genes that were identified in the main selected affected up-regulated or down-regulated pathways from CMA-dependent DEGs. Data show specifically the gene expression levels in KO PC cocultured with GB cells (relative to PC basal levels and normalized to β-actin as the housekeeping reference gene expression) and compared to WT PC co-cultured with GB cells. GB cells were used as negative control of gene expression (not shown). All data represent mean ± SD obtained from at least, five experiments using U373 and U87 GB cell lines, independently; *p < 0.05; **p < 0.01; ***p < 0.005. **(F)** Phagocytic capacity (Ph capacity) of KO PC (below) versus WT PC (above) against GB cells. Figure shows representative images of PC visualized with phalloidin (Phall-PC; green) displaying phagocytic activity by engulfing of pyknotic nuclei (with the DNA dye DAPI, white; arrows) and/or cytoplasmic inclusions (arrowheads) of apoptotic GB, stained with DiI and phalloidin (APO-GB; yellow), and compared to control PC without APO-GB (vehicle). Images are representative of five independent experiments of both GB cell lines (U373 and U87), using U373; Scale bars: 50 μm. **(G)** Graph represents percentage of PC showing phagocytic pouches (Ph capacity) from the total number of WT PC and KO PC in the absence (vehicle) or the presence of GB. Data show mean ± SD obtained from images of at least, five independent experiments using apoptotic U373 and U87 GB cell lines, independently; ***p < 0.005.

To reveal the gene pathways affected by GB-induced CMA in PC, we performed RNAseq studies to compare the differentially expressed genes (DEGs) between LAMP2A KO PC and WT PC, both in absence and in presence of GB. A total of 707 DEGs were detected between KO PC compared to WT PC in absence of GB, of which 478 genes were up-regulated (higher expression in deficient CMA PC) and 229 genes were down-regulated (Figure 1A). On the other hand, a total of 713 DEGs were also identified to be dependent of CMA but also of GB, of which 402 genes were up-regulated in KO PC + GB and 311 genes were down-regulated (Figures 1A,B). But most importantly, a total of 456 DEGs from those genes, were detected to not-overlap with just CMA-dependent DEGs between KO PC and WT PC. 249 genes were up-regulated in KO PC + GB and 207 genes were down-regulated (grey dark circles in Figure 1A).

DEGs were analyzed by the Gene Ontology enrichment to determine the affected biological pathways (Figure 1C). Kyoto Encyclopedia of Genes and Genomes (KEGG) pathway enrichment analysis from CMA-dependent DEGs revealed several gene expression pathways up- or down-regulated in CMA-deficient PC and affected by GB-induced CMA (Supplementary Figure S1A). Moreover, some of the most up-regulated and down-regulated genes of the selected CMA-dependent pathways (Supplementary Figure S1B) were corroborated by a heatmap (Figure 1D) and validated (Figure 1E).

In agreement with our previous findings, the main affected up-regulated pathways in KO PC in presence of GB were related to immune and inflammatory responses, and other anti-tumor cell functions such as cell-adhesion (Figure 1C, above and Supplementary Figure S1A, left), which is altered in CMA-deficient PC and prevents stable interaction with GB cells (Valdor et al., 2019; Molina and Valdor, 2020).

In addition, the network visualization of these affected CMA-dependent up-regulated pathways in KO PC showed large overlap between them (Figure 1C, above). On the contrary, the main affected down-regulated pathways such as angiogenesis, cell adhesion, regulation of actin cytoskeleton and others (Figure 1C, below and Supplementary Figure S1A, right) were related to the pro-tumoral functions observed in PC with GB-induced CMA (Molina et al., 2019; Molina and Valdor, 2020). Interestingly, the network visualization of the main affected CMA-dependent down-regulated pathways showed also an overlap between them with the exception of the angiogenesis and regulation of actin cytoskeleton pathways, which appeared independent from the others (Figure 1C, below).

Importantly, one of the most up-regulated CMA-dependent pathways that was identified in KO PC was the phagosome pathway (Figure 1C and Supplementary Figure S1B), which suggested an increase in the PC phagocytic activity as an anti-tumor function of KO PC. To support this finding, we confirmed the gene expression related to this pathway (Figure 1E) and we validated the protein expression of some of the most up-regulated genes (Supplementary Figures S1B, S2). Our results revealed that the catalytic subunit of the peripheral V1 complex of vacuolar ATPase (ATP6V1A) gen (Supplementary Figure S2A) and a cluster of major histocompatibility complex (MHC) class I and class II genes (Supplementary Figure S2B) were highly expressed in KO PC in presence of GB (KO PC + GB) when compared to GB-conditioned PC (WT PC + GB).

Furthermore, to validate phagocytic activity in PC, we compared the phagocytic capacity of WT and KO PC in co-cultures with apoptotic GB, as it has been previously reported (Diaz-Aparicio et al., 2016). Apoptotic cells, which have been previously characterized to express other apoptosis markers such as activated caspase 3 and fractin, were defined as pyknotic/karyorrhectic nuclei labeled with the DNA dye DAPI (Sierra et al., 2010). The PC phagocytic capacity (Ph capacity, i.e., the proportion of pericytes with one or more phagocytic pouches, each containing one apoptotic cell) (Sierra et al., 2010) was significantly increased in KO PC (Figures 1F, G and Supplementary Figure S3). Taking together all the previous results, the CMA ablation in PC in response to GB cells showed an expected immunogenic phenotype able to phagocyte GB cells and a key strategy to develop future therapies against GB.

### Differential Expression of Protein Secretion from Co-Cultures of GB with CMA-Deficient PC Reveals New Prognosis Markers for Tumor Progression

**FIGURE 2:**
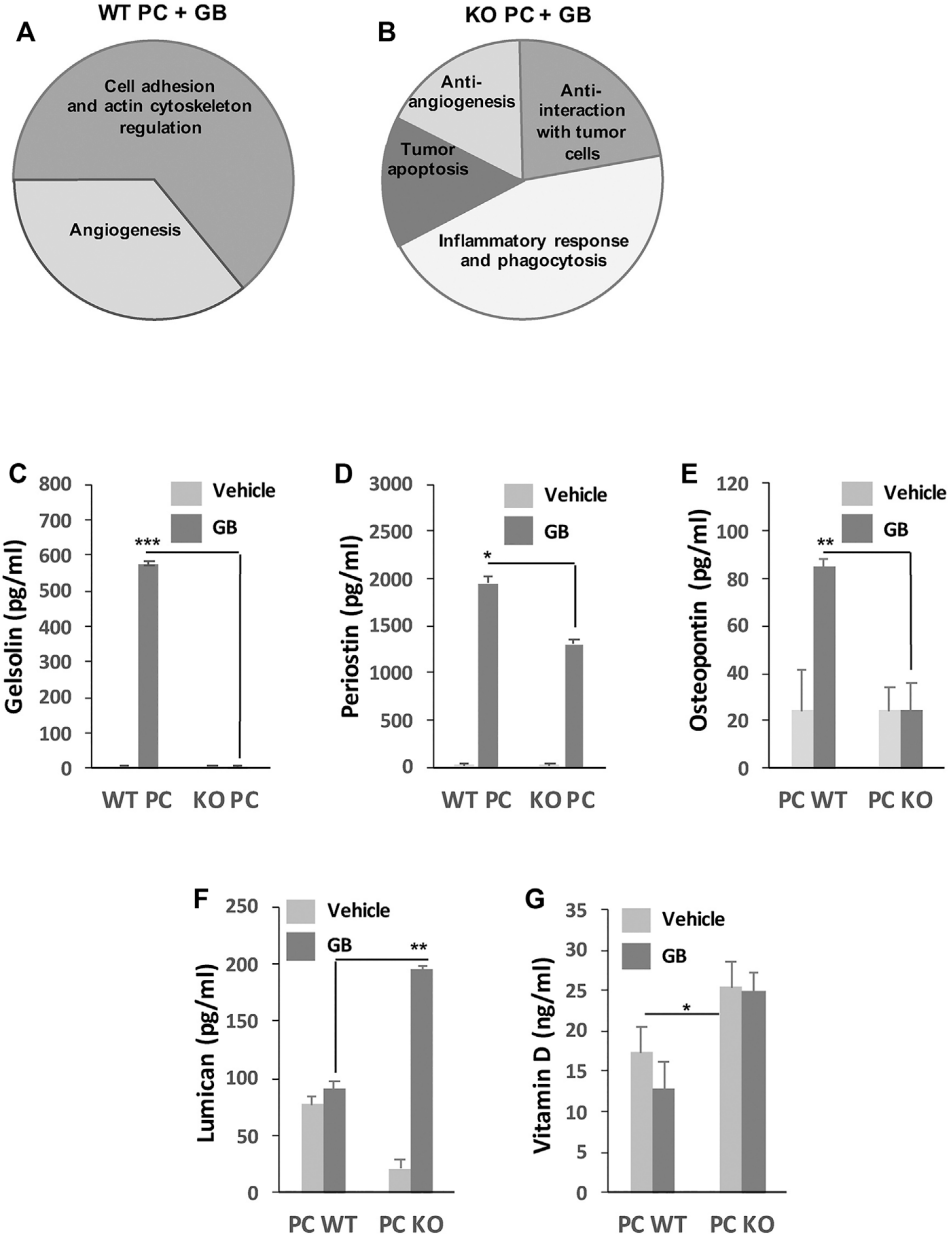
GB prognostic marker analysis. **(A,B)** General diagram shows the analysis of functional groups associated to pro-tumor **(A)** and anti-tumor **(B)** mechanisms. Those groups represent the percentage of proteins differently identified in the secretome of pro-tumor WT PC + GB **(A)** and anti-tumor KO PC + GB **(B)** co-cultures from the total amount of identified proteins by mass spectrometry and that were assocciated with pro-tumor and anti-tumor mechanisms. From the total amount of 31 identified proteins, 16 proteins were identified as preferentially secreted by WT PC + GB and 15 proteins preferentially secreted by KO PC + GB. 10 (62.5%) of the proteins secreted by WT PC + GB were related to cell adhesion and actin cytoskeleton regulation functions (such as gelsolin, periostin, osteopontin), and the rest (37.5%) were related to angiogenesis functions (such as osteopontin). On the other hand, 6 (46%) of the proteins secreted by KO PC + GB were related to inflammatory response and phagocytosis (such as Vitamin D-binding protein), three proteins (20%) were related to anti-interaction with tumor cells (such as lumican), and the rest were related to tumor apoptosis (17%) and anti-angiogenesis (17%). Data were obtained from three independent experiments using U87 GB cell line. **(C-G)** Validation by ELISAs of some proteins and related molecules that were identified to be associated with mechanisms of the tumor immune response. Concentrations of mouse gelsolin **(C)**, periostin **(D)**, osteopontin **(E)**, lumican **(F)**, and vitamin D **(G)** produced by WT PC and KO PC in the absence (vehicle) or the presence of GB for 72 h are shown. Data represent mean SD obtained from at least, five independent experiments using both GB lines U87 and U373, independently. Secretome from both human GB lines was used as negative control of detection; *p < 0.05, **p < 0.01.

Previously, we have found that abnormal upregulation of CMA is a mechanism by which GB cells elicit the immunosuppressive function of PC and stabilize GB-PC interactions necessary for tumor cell survival. CMA-deficient PC co-cultured with GB cells result in the secretion of proteins that reduce tumor cell survival through prevention and disruption of PC-GB interactions, leading to PC become into inflammatory cells that promote antigen-specific tumor immune responses (Valdor et al., 2019). In order to elucidate the specific contribution of the PC secretome dependent of GB-induced CMA to the tumor immune responses and tumor cell survival, we performed comparative-proteomic analyses of proteins secreted by GB-conditioned PC (WT PC + GB) and compared to CMA deficient PC in presence of GB (KO PC + GB). As a result, several functional groups associated with pro-tumor and anti-tumor mechanisms were identified in each condition, respectively (Figures 2A,B).

Qualitative analysis of the secretome content isolated from WT PC + GB and KO PC + GB identified 31 proteins associated with pro-tumor and anti-tumor mechanisms that were differentially identified in the secretome of anti-tumor KO PC + GB, compared with the pro-tumor WT PC + GB and vice versa (Figures 2A,B). Thus, according to Biological Processes of Gene Ontology, 16 proteins were identified as proteins that may be preferentially secreted by pro-tumor WT PC + GB and that are related to pro-tumor mechanisms. 10 of them (62.5%) were related to cell adhesion and actin cytoskeleton regulation functions. The remaining proteins (37.5%) were related to angiogenesis functions (Figure 2A). All of them were related to the pro-tumoral immune function of GB-conditioned PC (WT PC + GB) previously reported (Valdor et al., 2019) and in concordance with the affected cell pathways that were detected (Figure 1).

As a result, data mining identified a subset of those proteins that are associated with the tumor immune responses (Figure 2A). Of those proteins, we focused our attention on different proteins, such as gelsolin (Giampazolias et al., 2021), periostin (Zhou et al., 2015; Huizer et al., 2020) and osteopontin (Wei et al., 2019; Li et al., 2021), which were exclusively detected in the secretome of WT PC + GB, and not in the other conditions. As these proteins might be good markers for GB prognosis dependent on GB-induced CMA in immunosuppressive PC (Valdor et al., 2019), we experimentally confirmed the presence of these proteins and their mouse origin in the secretome of GB-conditioned PC. Interestingly, our results revealed that all these proteins were dependent on GB-induced CMA (Figures 2C–E).

On the other hand, different protein fractions assocciated with anti-tumor mechanisms were identified in the secretome of KO PC + GB (Figure 2B). According to Biological Processes of Gene Ontology, of 15 proteins, six of them (46%) were related to inflammatory response and phagocytic activity, three proteins (20%) were related to anti-interaction with tumor cells and the rest were related to tumor apoptosis (17%) and anti-angiogenesis (17%). All identified functional groups were related to the anti-tumoral immune function of KO PC in presence of GB (Molina et al., 2019; Valdor et al., 2019) and in concordance with the affected pathways detected (Figure 1).

Of those functional groups, we focus our attention on the subset of proteins associated with molecules related to the immune response (Figure 2B). Then, we decided to determine the secretion levels of some of these interesting molecules (Figures 2F,G) that might be good prognosis markers for GB and indicate good outcome dependent on the anti-tumor immune function of PC and therefore, on the prevention of the tumor immune scape (Molina et al., 2019; Valdor et al., 2019). Lumican, a matrix protein which have an anti-tumor role inhibiting immune scape or even reversing several metastatic features in cancer cells (Karamanou et al., 2020; Giatagana et al., 2021; Zang et al., 2021) was produced in all experimental conditions, but mainly by KO PC in presence of GB as CMA dependent anti-tumor protein (Figure 2F). Instead, the secretion levels of vitamin D, as an anti-tumor molecule associated with immune responses (Zhang et al., 2019; Negri et al., 2020; Lo et al., 2021), were CMA-dependent in PC, but not GB-dependent since its secretion level was higher in KO PC, regardless of whether GB is present or not (Figure 2G). Thus, this last finding was not only useful for the prognosis of GB progression dependent of CMA, but even as a fact to subsequently develop therapeutical strategies against GB using CMA-deficient PC.

### Exofucosylated PC with Deficient CMA were Efficient to Reach the Tumoral Niche and Eliminate the Tumor Cells by Intravenous Therapy

**FIGURE 3:**
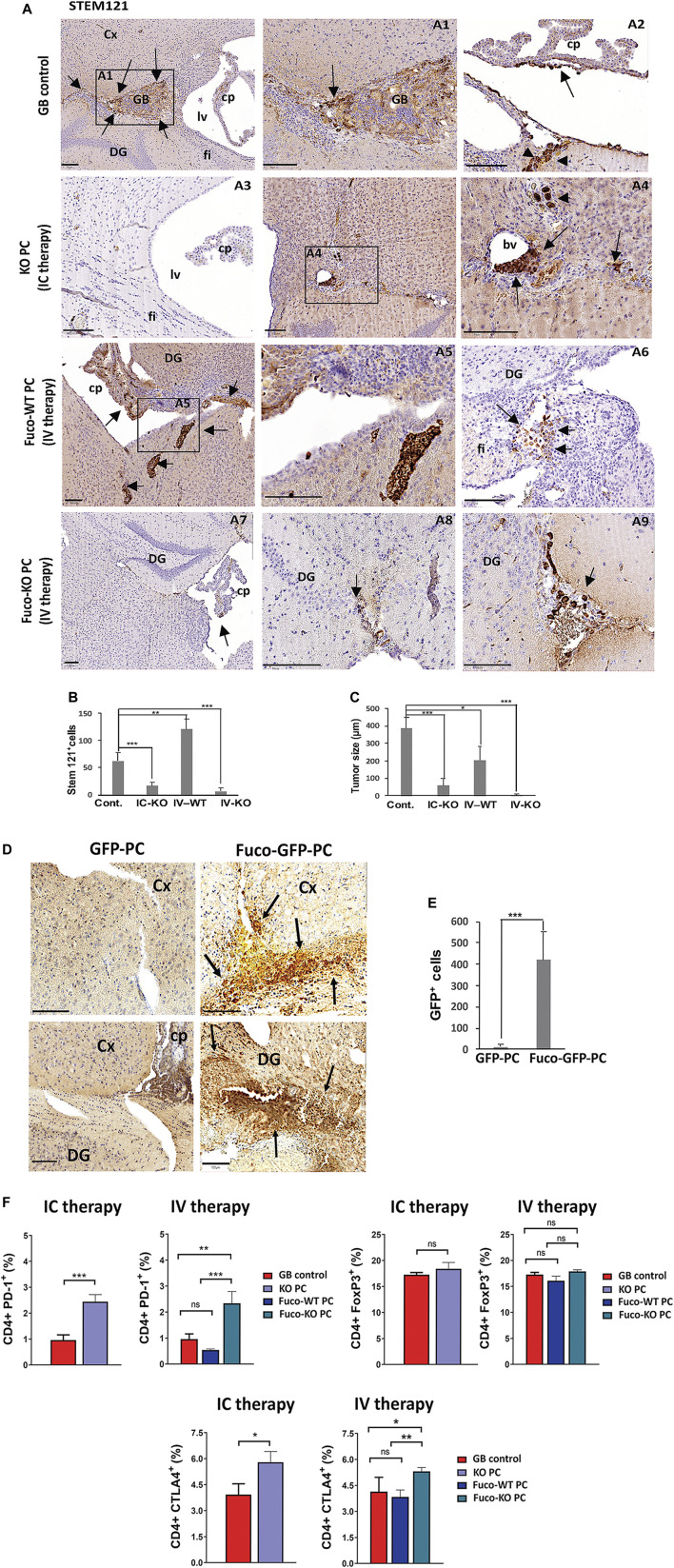
**(A)** GB proliferation in xenografts from mice treated with different strategies (IC therapy: Intracranial therapy with KO PC; IV therapy: Intravenous therapy with exofucosylated (Fuco) WT or KO PC after 1 month of GB tumor proliferation (GB control). GB cells were stained with STEM121 antibody. Arrows show tumor formation (GB control) around perivascular areas (arrow in A1) of the brain cortex (Cx) and infiltration of tumor cells in choroid plexus (arrow in A2) and subpial vessels (arrowheads in A2). The KO PC IC therapy shows a representative image (A3) of the grafted brain areas without rest of tumor and just some few tumor cells close to a blood vessel (bv; arrows in A4), graft trajectory and subpial meninges (arrowheads in A4). Representative images of meningeal tumor infiltration in a Fuco-WT PC IV therapy, tumor cell proliferation in choroid plexus (cp) and the brain parenchyma (arrows A5), tumor cells populated the most caudal part of the hypocampus and dentate gyrus (DG) and fimbria (fi; A5,6). Some rest of tumor cells of a previous tumor outside of the brain parenchyma were hardly detected in choroid plexus, perivascular and subpial regions of DG with the Fuco-KO PC IV therapy (arrows in A7,9); Scale bars: 100 μm. All results are shown using the U87 GB line, 4 weeks after therapy, and are representative of at least, three independent experiments using both U87 or U373 GB cell lines, independently. **(B)** Relative quantification of tumor cells related to the number of immunopositive stem 121 cells per brain **(C)** Morphometric measurement reveals the average of tumor size related to the surface area of the tumor cell mass as an irregular shape in GB control mice (six tumor masses/five grafted mice), GB mice intracranially treated with KO PC (IC-KO; three tumor masses/five grafted mice), GB mice treated with Fuco-WT (IV-WT; 13 tumor masses/five grafted mice or KO PC (IV-KO; 0 tumor masses/five grafted mice). All results are mean+SD from at least, three independent experiments using both U373 and U87 GB lines, independently; *p < 0.05, **p < 0.01, ***p < 0.001. **(D)** Grafts of exofucosylated GFP-PC that were injected intravenously in the mice grafted with U87 GB cells previously, were compared to those from the GB mouse model just treated with GFP-PC and stained with an anti-GFP antibody (Cx, brain cortex; cp, choroid plexus; DG, Dentate gyrus; Scale bars, 100 μm). **(E)** Relative quantification of the number of fucosylated PC (Fuco-GFP-PC) that reach tumor areas after therapy, compared to control (GFP-PC) and related to the number of GFP immunopositive particles. All results are mean+SD from at least, three independent experiments using both U373 and U87 GB lines, independently. ***p < 0.001. **(F)** Expression by flow cytometry of the negative regulators of T cell activation PD-1, FoxP3 and CTLA4 in central lymph nodes. Data represent mean ± SD obtained from at least, three independent experiments using both GB lines U87 and U373, independently. *p < 0.05, **p < 0.01, ***p < 0.001.

We have previously seen that GB-induced CMA in PC assists tumor growth *in vivo* through GB-PC interactions and failed anti-tumor T cell responses (Valdor et al., 2019). The lack of CMA in PC with GB prevents PC-GB interactions, allowing the secretion of proteins that reduce tumor cell survival and the acquisition of an immunosuppressive function in PC following tumor interaction (Valdor et al., 2019). To determine if CMA ablation in PC affects its ability to promote tumor cell elimination in the GB tumoral niche, we analyzed the tumor growth in our xenograft mouse model of GB after intravenous administration of exofucosylated WT and KO PC and/or after intracranial administration of KO PC compared to tumor growth observed in control mice (Figures 3A–C). As expected, GB control mice, showed brain infiltration of tumor cells in choroid plexus and subpial vessels, and tumor masses in brain cortex around perivascular areas. In contrast, GB control mice that were grafted with KO PC, showed only a few tumor cells close to blood vessels with no other evidence of tumor mass, after 1 month of therapy (IC, intracranial therapy; Figures 3A–C). Corroborating previous results (Valdor et al., 2019), higher GB progression in the brain parenchyma and solid tumor mass and infiltration were observed in the GB mouse model treated with exofucosylated WT PC (Fuco-WT PC; IV, intravenous therapy) compared to control mice and the other strategies. In contrast, cells of a previously engrafted tumor were hardly detected outside of the brain parenchyma in the GB mouse model after being treated with exofucosylated KO PC (Fuco-KO PC; IV; Figures 3A–C). Although the difference in results after therapy with Fuco-WT/KO PC was obvious compared to intracranial therapy, we wanted to demonstrate that exofucosylated PC (Supplementary Figure S4) were able to reach the tumor niche, going through the blood brain barrier (BBB). For this, GB-grafted mice were intravenously injected with Fuco-GFP-PC and compared to those injected with control unmodified GFP-PC. Fuco-GFP-PC infiltrates in peritumoral areas and around blood vessels of the brain cortex and dentate gyrus were detected after 1 week of treatment (Figures 3D,E). Fuco-GFP-PC distribution was maintained until 5 days later and such as we previously described with GB-conditioned PC intracranially grafted (Valdor et al., 2019). However, after that time, PC were hardly detected (not shown).

As expected, flow cytometry analyses of CD4^+^ T cells from central draining lymph nodes of the GB mice treated with different strategies showed that either intracranial or intravenous therapy with KO PC seem to be effective to activate the anti-tumor T cell responses. After therapy with KO PC, T cells presented significant higher levels of PD-1 and CTLA-4, two inhibitory T cell receptors that are present in activated T cells, exhausted T cells and some subsets of memory T cells (Fuertes Marraco et al., 2015; Morris et al., 2018), whereas the levels of the T regulatory cells (Tregs) expressing transcription factor FoxP3 was not affected in any of the therapies (Figure 3F).

**FIGURE 4:**
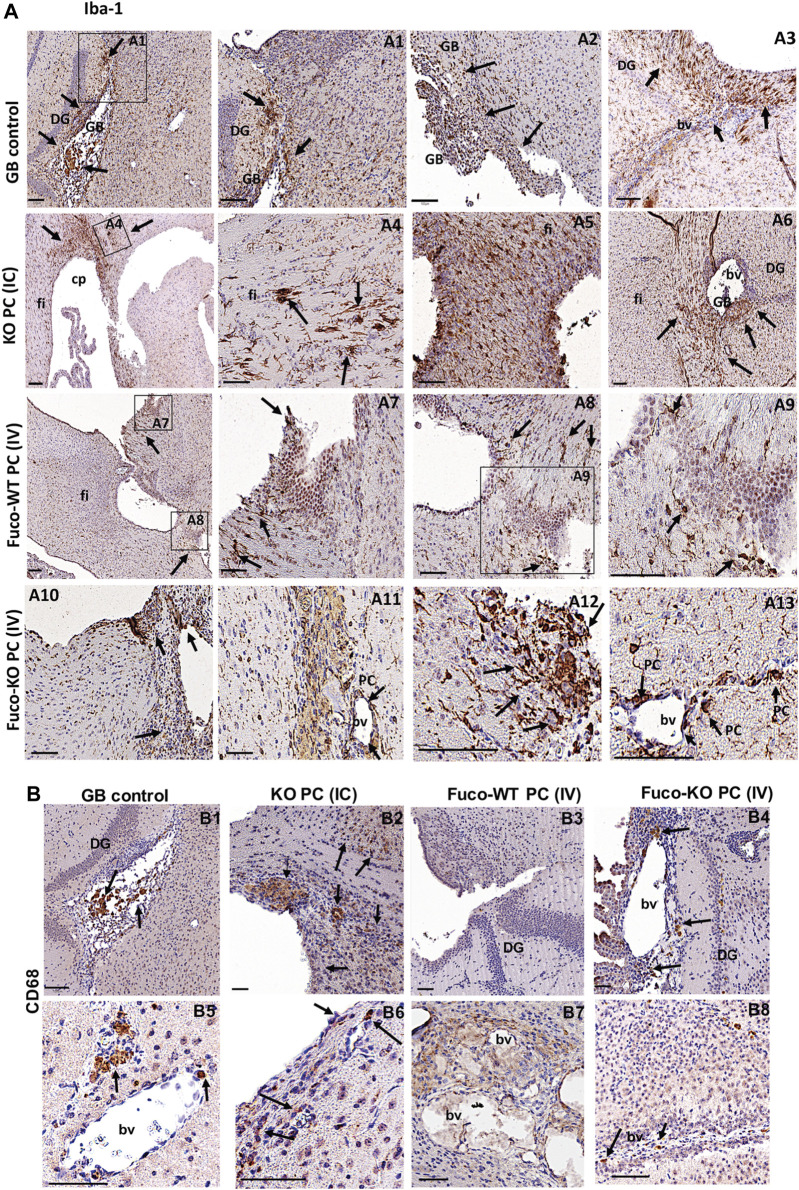
Expression of the phagocytic markers Iba-1 **(A)** and CD68 **(B)** in GB xenografts from mice treated with different therapeutic strategies (Scale bar, 100 μM). Arrows in **(A)** show abundant Iba-1 immunopositive cells in the tumor (arrows A1,2) and host peritumoral brain parenchyma (arrows in A1-3). IC therapy show a big accumulation of microglia activated in areas where there was a previous tumor that was eliminated (arrows in A4-6) or in perivascular areas where there is still some tumor cells debris (arrowheads in A6). The Fuco-WT PC IV therapy just show rest of activated microglia around tumor cell areas (arrows in A7-9). The Fuco-KO PC IV therapy shows accumulation of activated microglia (arrows in A10-13), some activated pericytes (PC in A11, 13) and some infiltrated blood cells such as activated macrophages (arrows in A10-13) in along perivascular areas and close to previous tumorigenesis that was eliminated. **(B)** The immunostaining for CD68 was only positive in some blood cells and perivascular cells with the GB control therapy (B1,5) and mice treated with (IV) Fuco-WT PC and KO PC (B3,7, 4, 8). But was very reactive in the affected brain parenchyma and perivascular areas of mice treated with the KO PC IC therapy (B2,6). All shown results are representative of at least, three independent experiments using both lines GB U373 and U87, independently.

In agreement with previous results on the CMA-dependent phagocytic capacity in PC and their ability to modulate inflammation depending on GB-induced CMA (Figures 1, 2), we also found that depending on the therapy type different phagocytic cell populations are contributing to the tumor clearance in the anti-tumor innate immune response (Figure 4 and Supplementary Figures S5A–C). The microglia activation marker Iba-1, also expressed in activated PC and macrophages (Sakuma et al., 2016; Wallet et al., 2019) was found expressed in grafts of the GB control mouse model, showing gliosis accumulation in the tumor and peritumoral areas. Grafts from mice treated with the KO PC IC therapy showed a significant accumulation of activated microglia in previous tumor areas and some cell debris in perivascular areas, where, there was still some remnant tumor cells (Figure 4A and Supplementary Figures S5A,B). Excitingly, the Fuco-KO PC IV therapy showed great immunoreaction for Iba-1 in microglia, perivascular cells and infiltrated blood cells along perivascular areas and close to previous tumorigenesis that was eliminated (Figure 4A and Supplementary Figures S5A,B). However, Iba-1 immunopositive cell debris were hardly observed around tumor cell areas after the Fuco-WT PC IV therapy (Figure 4A and Supplementary Figures S5A,B). Interestingly, the macrophage activation marker CD68, also expressed in some cases in activated PC and microglia (Navarro et al., 2016; Shibahara et al., 2020; Sun et al., 2021), was highly expressed just in grafts of the mice treated with the KO PC intracranial therapy (Figure 4B and Supplementary Figure S5C).

## Discussion

The facts that glioma stem cells resist conventional treatments and the lack of current effective therapies (Arvanitis et al., 2020; Sampson et al., 2020), raise the urgent need for new treatments in GB (Molina et al., 2019; Jacob et al., 2020).

We have previously described a potential effective strategy to eliminate the GB tumor growth through modulation of CMA in brain PC (Valdor et al., 2017; Molina et al., 2019; Valdor et al., 2019; Molina and Valdor, 2020). The modulated CMA promotes the secretion of toxic proteins that eliminate tumor cells, restores mesenchymal stem cell like-properties, and improves changes in the immune properties of PC that avoid tumor progression (Valdor et al., 2019). Furthermore, our GB mouse model, grafted with CMA-deficient PC at the initiation of the tumor formation, escapes from the state of immunosuppression caused by tumor cells. In addition, it shows reduced tumor cell proliferation and survival compared to control mice (Valdor et al., 2019).

With our work, we wanted to identify new biomarkers and altered functions to get more insights in the pathological dysregulation of GB-induced CMA in PC that assists pro-tumor immune responses and GB progression. Furthermore, we also proposed to further characterize in the PC biology dependent on CMA, if CMA-deficient PC might be considered a new cell-therapy strategy against GB.

Our data shows GB-dependent great alterations in gene expression in PC, revealing specific PC functions dependent on GB-induced CMA, and, therefore, specific targets useful for future therapies (Figures 1A–E). Our results agrees with our previous findings (Valdor et al., 2019; Molina and Valdor, 2020), as the main affected up-regulated pathways in CMA-deficient PC in response to tumor cells, were related to immune and inflammatory responses, and other cell functions such as cell-adhesion (Figure 1C, above and Supplementary Figure S1A, left). The affected down-regulated pathways, such angiogenesis, cell adhesion, regulation of actin cytoskeleton and others (Figure 1C, below and Supplementary Figure S1A, right), corroborated the previously observed pro-tumoral functions that GB induces on PC through aberrant upregulation of CMA. Interestingly, the DEGs in CMA-deficient PC revealed overlap between some cell pathways except from the angiogenesis and regulation of actin cytoskeleton pathways (Figure 1C). Angiogenesis function in PC seem to be the most affected by GB-induced CMA independently of other affected PC functions, and might be very important to target specific genes for future therapies without affecting other cell functions. In addition, the fact that PC act as mediators of angiogenesis in other diseases (Carmeliet and Jain, 2011; Sakuma et al., 2016) suggests that the modulation of CMA in PC in response to other stressors different to GB, might be a specific and good target for angiogenesis-related therapies.

Importantly, one of the most up-regulated CMA-dependent pathways validated in CMA-deficient PC was the phagocytic function (Figure 1E and Supplementary Figures S1B, S2). This result underlies an expected immunogenic phenotype against the tumor cells able to activate the specific anti-tumor T cells responses (Fuertes Marraco et al., 2015; Nakashima et al., 2018; Valdor et al., 2019). The fact that deficient CMA-PC are also able to phagocyte apoptotic tumor cells (Figures 1F,G and Supplementary Figure S3) indicates that not only glial cells are implicated in the clearance of tumor debris, but PC are active phagocytic cells that also contribute to the antigen-specific T cell responses (Hutter et al., 2019; Valdor et al., 2019). As a possibility, the anti-tumor T cell responses might be activated by PC, through the antigen presentation in response to phagocytosis of immunogenic tumor vesicles or tumor fragments (Dionisi et al., 2018; Li et al., 2018; Morris et al., 2018). They might also be activated in response to PC phagocytosis of whole tumor cells, which could facilitate the antigen presentation to CD4^+^ T cells as occurs with some professional antigen-presenting cells (von Roemeling et al., 2020; Caronni et al., 2021). In addition, the increased protein expression of some genes associated with the phagosome function (Supplementary Figure S2) also supports the immunogenic phenotype of CMA-deficient PC. The results suggest that their high expression in KO PC might be associated with the acidification of phagosomes or other cell compartments (Sun-Wada et al., 2009; Banerjee and Kane, 2020; Vasanthakumar and Rubinstein, 2020), that contribute to antigen processing and presentation, and therefore, to the tumor immune responses (Ackerman et al., 2003; Mantegazza et al., 2013; Das Mohapatra et al., 2020; Kozik et al., 2020; Dhatchinamoorthy et al., 2021).

Previously, we have shown that CMA-deficient PC co-cultured with GB cells result in increased secretion of proteins that reduce tumor cell survival both preventing PC-GB interactions and disrupting the pre-established ones (Valdor et al., 2019). In this work, we have identified different functional groups related to pro-tumor mechanisms from enriched protein fractions in the secretome of pro-tumoral WT PC + GB (Figure 2A). Previous reports support the pro-tumoral role in the immune response of some identified proteins secreted by GB-conditioned PC (Figures 2C–E). Secreted gelsolin is linked to the inhibition of anti-tumor immune mechanisms and tumor immunity (Giampazolias et al., 2021). Periostin is a matrix protein that recruits pro-tumoral macrophages and enhances GB progression in mice (Zhou et al., 2015), and recently, it has been reported to be expressed by PC in gliomas, favoring angiogenesis (Huizer et al., 2020). Finally, osteopontin has been described as a potent chemokine recruiting macrophages to GB tumor cells and mediating crosstalk between tumor cells and the failed anti-tumor immune response (Wei et al., 2019). Our results show that gelsolin, periostin and osteopontin might be good candidate biomarkers for a poor outcome during GB cancer progression dependent on GB-induced CMA in PC.

Besides, our data show potential biomarkers for good GB prognosis such as lumican (Karamanou et al., 2020; Giatagana et al., 2021; Zang et al., 2021) and vitamin D (Zhang et al., 2019; Negri et al., 2020; Lo et al., 2021) (Figures 2F,G) in the identified subset of the CMA-deficient PC secretome with GB, including proteins related to anti-tumor mechanisms and immune responses (Figure 2B). Vitamin D has been associated with reduced risk of cancer death (Zhang et al., 2019), to be an immunomodulatory factor that potentiate cancer therapy by reversing drug-resistance in cancer cells (Negri et al., 2020; Ao et al., 2021), and to have anti-tumor effects in GB (Lo et al., 2021). However, the secretion levels of vitamin D in PC, were CMA-dependent, but not GB-dependent (Figure 2G). Therefore, our data indicates that the use of CMA-deficient PC, as anti-tumor cells secreting toxic molecules for tumor survival that modulate inflammation and tumor immune responses, may be a potential tool to develop therapeutical strategies against GB.

Considering the anti-tumor properties of CMA-deficient PC against GB, and based on our previous studies of grafted PC with normal CMA activity (Valdor et al., 2019), we performed experimental strategies in our GB mouse model, using CMA-deficient PC to eliminate tumor cells (Figures 3A–C). As we had shown that PC brain grafts are not detected in an immunocompetent mouse model compared to PC grafts previously conditioned by GB (Valdor et al., 2017), we proposed as a new therapeutic strategy, treating GB mice with WT PC or KO PC intravenously (Figures 3A–C). On the other hand, PC display mesenchymal stem cell like-properties and it has been reported that mesenchymal stem cells modified by enzymatic exofucosylation ensures effective cell migration to affected areas with an up-regulated expression of E-selectin within tumor endothelial beds. Modified mesenchymal stem cells present important differences between the pattern of soluble molecules secretion compared to unmodified counterparts, including cytokines and chemokines with pro-inflammatory function (Sackstein et al., 2008; Abdi et al., 2015; Garcia-Bernal et al., 2020). Thus, we exofucosylated the PC as a cell-engineering tool to increase the PC recruitment to brain affected areas after tumor establishment. Remarkably, our results show that the PC exofucosylation is successful to make them reach the tumor areas in the brain parenchyma (Figures 3D,E and Supplementary Figure S4), and this could be very useful for future cell therapies not only for GB cancer but for other diseases. Although, both strategies using CMA-deficient PC (intracranial and intravenous) were useful to reduce tumor cell progression and, likely tumor clearance by anti-tumor T cell responses (Figure 3F) (Valdor et al., 2019), our data indicate that the intravenous therapeutic strategy with exofucosylated CMA-deficient PC is more efficient to eliminate the GB tumor (Figures 3A–C). Furthermore, in agreement with previous results on the CMA-dependent phagocytic capacity in PC (Figures 1F,G and Supplementary Figure S3), this strategy also activated different phagocytic cell populations (Figure 4 and Supplementary Figure S5), including PC (Supplementary Figure S5B), that can contribute to the anti-tumor innate response (Sakuma et al., 2016; Wallet et al., 2019). Compared to other strategies (Figure 4B and Supplementary Figure S5C), we could also detect some blood cells infiltration and perivascular cells with high expression of the CD68 phagocytic marker related to brain damage (Navarro et al., 2016; Shibahara et al., 2020; Sun et al., 2021). But compared to the intracranial one, our results suggest that this strategy may be the less aggressive for future clinical therapies.

In summary, our data corroborate our previous findings on the impaired immunogenic function of PC with GB-induced CMA, driving to additional altered PC functions and also to the identification of new biomarkers related to the tumor immune responses for GB prognosis/therapy. This work demonstrates CMA ablation in PC as a key target mechanism to develop new potential therapies against GB tumor progression.

## Data Availability

The original contributions presented in the study are publicly available in ENA under accession number PRJFB48545.
