# Supplementary figures and images for: Chaperone-Mediated Autophagy Ablation in Pericytes Reveals New Glioblastoma Prognostic Markers and Efficient Treatment Against Tumor Progression
Source: Front Cell Dev Biol. 2022 Mar 18;10:797945. doi: 10.3389/fcell.2022.797945 (PMC8997287; doi:10.3389/fcell.2022.797945)

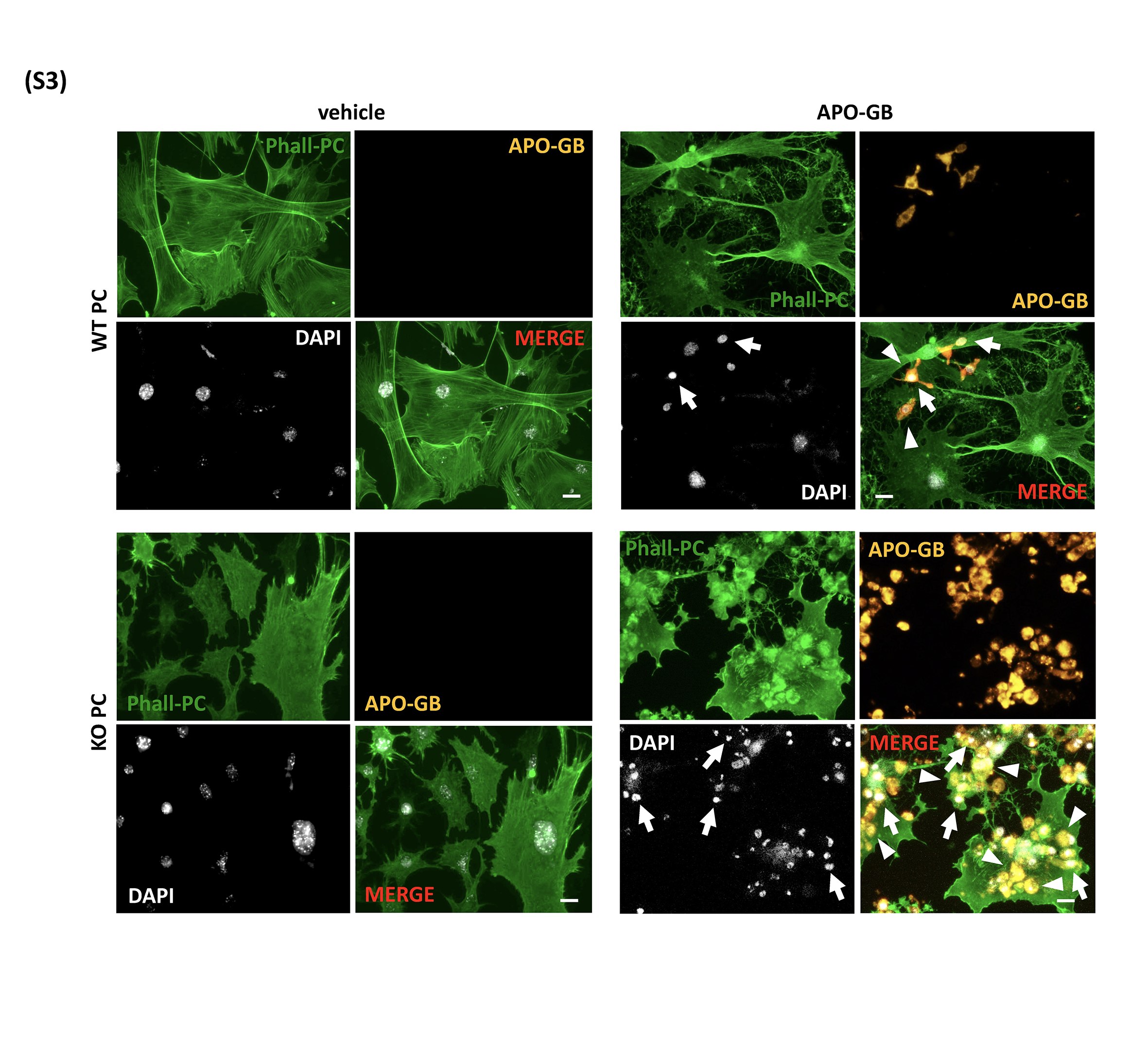

Supplement: Supplementary file 1 [file Image3.TIF]

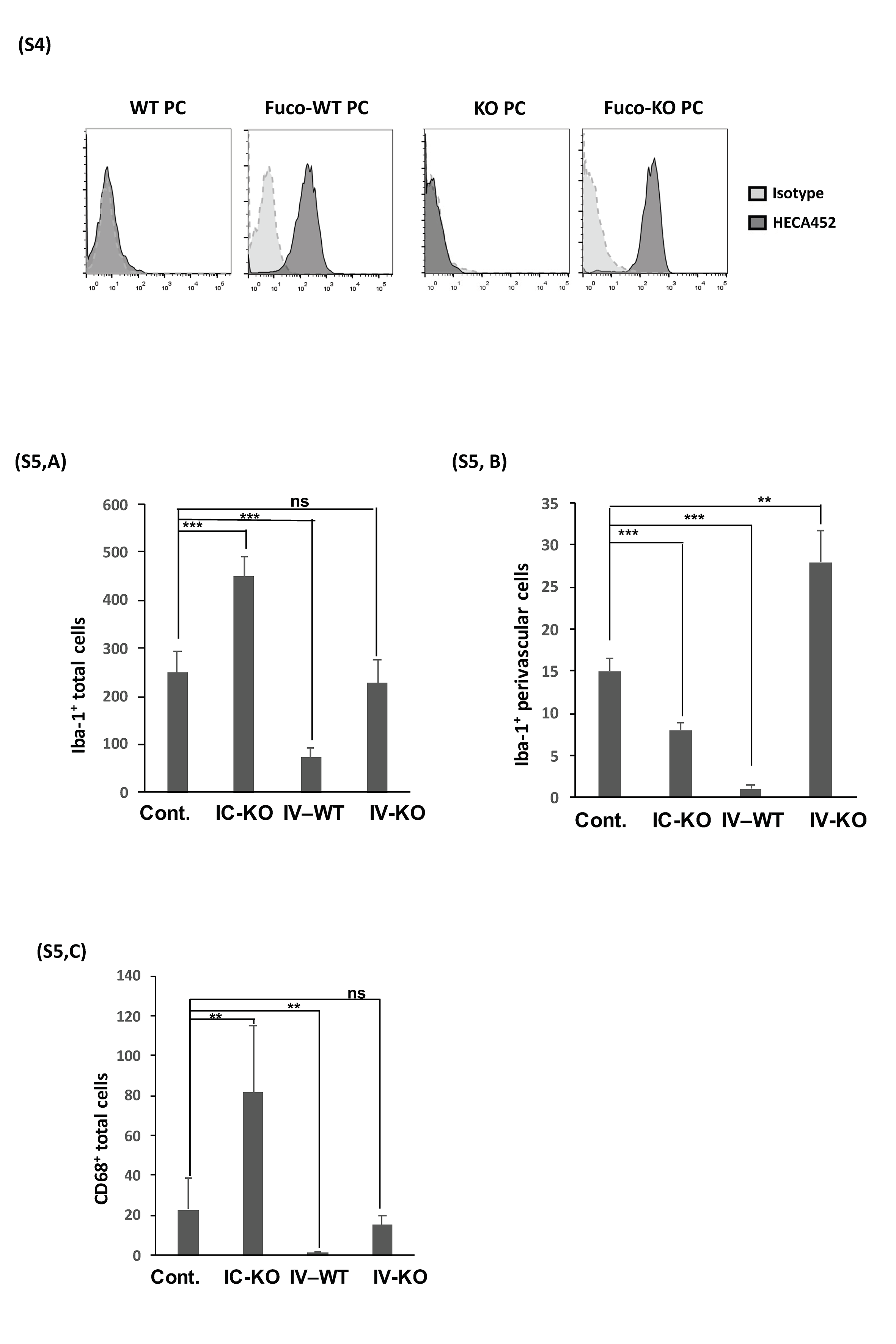

Supplement: Supplementary file 2 [file Image4.TIF]

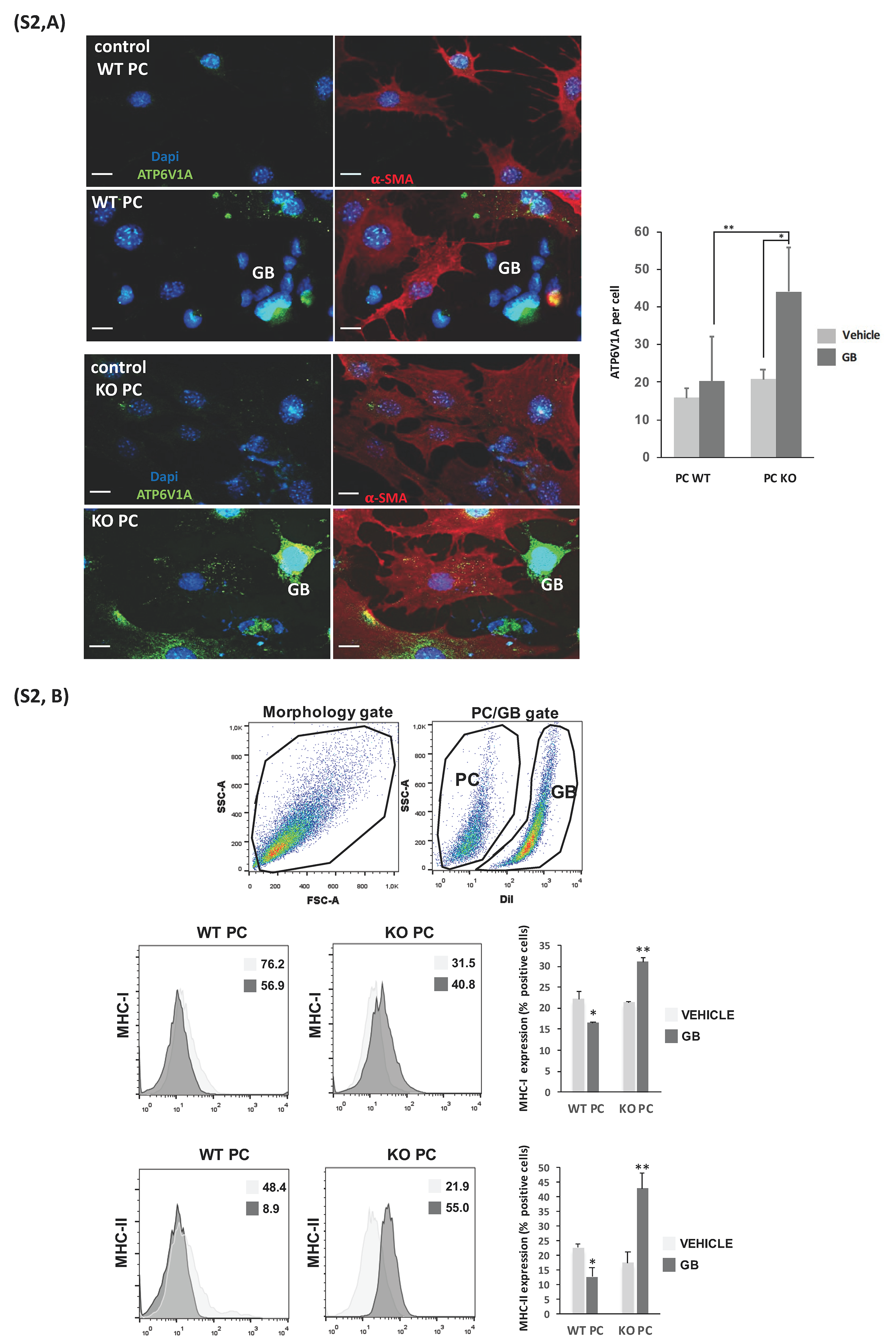

Supplement: Supplementary file 3 [file Image2.TIF]

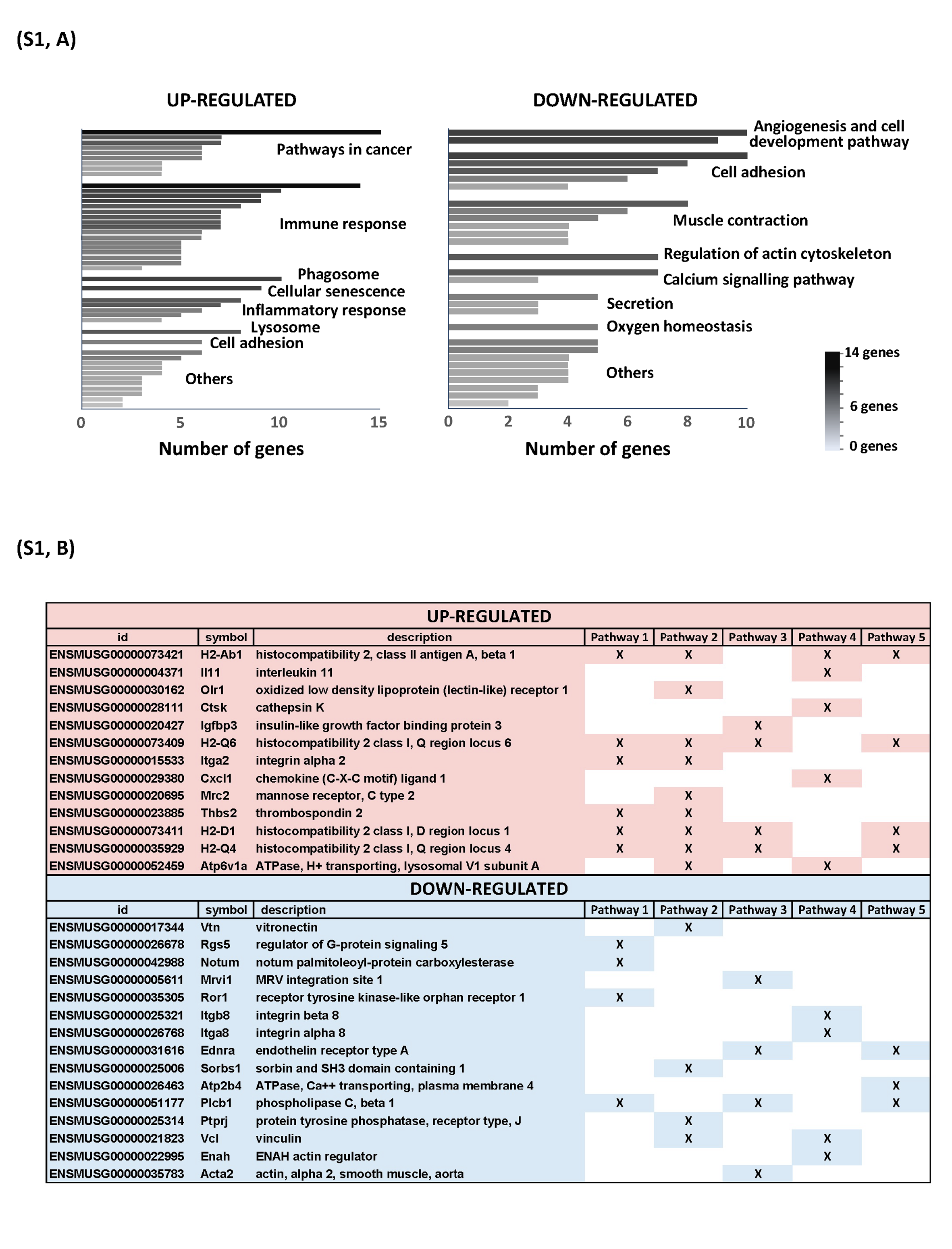

Supplement: Supplementary file 4 [file Image1.TIF]
