# Supplementary material for: Chaperone-Mediated Autophagy Ablation in Pericytes Reveals New Glioblastoma Prognostic Markers and Efficient Treatment Against Tumor Progression
Source: Front Cell Dev Biol. 2022 Mar 18;10:797945. doi: 10.3389/fcell.2022.797945 (PMC8997287; doi:10.3389/fcell.2022.797945)
Supplement: Supplementary file 5 [file DataSheet1.pdf]

frontiers

Supplementary Material

1 Supplementary Data

Supplementary Material and methods

RNA sequencing and differential expression analysis. Libraries were sequenced to obtain more than 50 million reads in total (WT1: >49.9M; WT2: >49.1M; WT3: >53.0M; KO1: >50.6M; KO2: >51.5M; KO3: >51.9M; WTGB1: >104.9M; WTGB2: >103.3M; WTGB3: >52.6M; KOGB1: >116.7M; KOGB2: >112.2M; KOGB3: >53.8M). Quality control of sequenced reads was performed using FastQC v0.11.8 (Babraham Institute). Sequencing reads were aligned using HISAT2 v2.1.0 (Kim et al., 2015) to GRCm38/mm10 mouse genome index masked with the mouse Single Nucleotide Polymorphism Database (dbSNP) (Sherry et al., 1999) build 142. Aligned reads numbers per library were as follows: WT1: >47.5M; WT2: >46.8M; WT3: >50.5M; KO1: >47.7M; KO2: >49.4M; KO3: >49.4M; WTGB1: >20.0M; WTGB2: >9.44M; WTGB3: >15.6M; KOGB1: >26.2M; KOGB2: >12.5M; KOGB3: >17.4M. Sequencing reads of mixed cultures (murine PC with human GB) were further aligned using HISAT2 v2.1.0 to GRCh38/hg38 human genome index masked with human dbSNP build 151. Aligned reads numbers per library were as follows: WTGB1: >40.0M; WTGB2: >47.9M; WTGB3: >37.5M; KOGB1: >41.8M; KOGB2: >47.1M; KOGB3: >36.8M. Reads overlapping annotated genes (mouse, Ensemble GRCm38.95; human, Ensemble GRCh38.95) were counted using HTSeq v0.11.1 (Anders et al., 2015).

KEGG pathway enrichment analysis (Figure S1). KEGG pathway enrichment analysis has been performed with both the up-regulated and down-regulated genes (independently). For this, the package limma (Ritchie et al., 2015) of R (R Core Team, 2021) has been used. Subsequently, the enrichment results have been grouped according to the pathway.

Real-Time PCR (qPCR). To validate the expression of up-regulated or down-regulated genes, the following primers (Sigma-Aldrich) were used: mouse H2D1 5′-GATGAAGAGAAGGAGAAACAC-3′ and 5′-GTCTTCACGCTTTACAATCTC-3′, mouse H2Ab1 5′-GGAGTCAGAAAGGACCTC-3′ and 5′-TCTGAGACAGTCAACTGAG-3′; mouse Olr1 5′-GAACGAGAAATCCAAAGAGC-3′ and 5′- ACAAGGACCTGAAAAGTTTG-3′; mouse Thbs2 5′-CACCATCAGTGAACATACAG-3′ and 5′-GTTATCACTAGACACTCTCTCC-3′; mouse Mrc2 5′-CTGTGAACTATTCTAACTGGG-3′ and 5′-CTGTTCTCTTCCACTCTAGG-3′; mouse Atp6v1a 5′-AGGTTGATGGGAAAGTAGAG-3′ and 5′-AATTCCAGTGTAGATGGAGG-3′; mouse Vtn 5′-TACTTGTTCAAGGGTAGTCAG-3′ and 5′-ATCAACATTGTCTGGTATGC-3′; mouse Rgs5 5′-CCAAGGAGATCAAGATCAAG-3′ and 5′-GCAAATCCATAGCTGTTCTG-3′; mouse Acta2 5′-GTCCCAGACATCAGGGAGTAA-3′ and 5′-TCGGATACTTCAGCGTCAGGA-3′; mouse (5’-AAGGACTCCTATGTGGGTGACGA-3’; 5’- ATCTTCTCCATGTCGTCCCAGTTG-3’) and human (5’-GTCTGCCTTGGTAGTGGATAATG-3’; 5’-TCGAGGACGCCCTATCATGG-3’) β-Actin expression were measured as reference housekeeping controls.

Secretome analysis:

Sample complexity negatively affects the ability to detect, identify and quantify low-abundance proteins by mass spectrometry (MS) analysis since peptides from high-abundance proteins can mask

detection of those from low-abundance proteins (Pascovici et al., 2016). Thus, for sample preparation, we use *Blue*Prep Major Serum Protein Removal Kit (SERVA) following manufacturer´s recommendations. Control GB media, control cell culture media, cell culture media from WT PC or KO PC co-cultured with and without GB, were depleted from the six major serum proteins through chromatography spin-columns (SERVA). Subsequently, the proteins of the cell culture media were quantified and digested with trypsin, identified by means of HPLC-MS/MS analysis and validated using auto thresholds by the Proteomics facilities of the University of Murcia-IMIB Arrixaca.

In-solution trypsin digestion. Samples were digested with the following standard procedure. Samples were dissolved in 100 µl of 50 mM ammonium bicarbonate buffer pH 8.5 containing 0.01% ProteaseMax (Promega). This surfactant enhances the trypsin digestion. Protein samples were reduced by adding 20 mM DTT at 56°C for 20 min. Then, samples were alkylated by adding 100 mM IAA during 30 min at room temperature in the dark. Finally, digestion was performed by adding 1 µg of Trypsin Gold Proteomics Grade (Promega) (1:100 w/w) for 3h at 37 °C. Reaction was stopped with 0.1% formic acid and filtered through 0.22 µm. Finally, samples were dried using an Eppendorf Vacuum Concentrator model 5301.

HPLC-MS/MS analysis. The separation and analysis of the tryptic digests of the samples were performed with a HPLC/MS system consisting of an Agilent 1290 Infinity II Series HPLC (Agilent Technologies) equipped with an Automated Multisampler module and a High Speed Binary Pump, and connected to an Agilent 6550 Q-TOF Mass Spectrometer (Agilent Technologies) using an Agilent Jet Stream Dual electrospray (AJS-Dual ESI) interface.

Dry samples from trypsin digestion were resuspended in 20 µl of buffer A, consisting in water/acetonitrile/formic acid (94.9:5:0.1). Samples were injected onto an Agilent AdvanceBio Peptide Mapping HPLC column (2.7 µm, 150 × 1.0 mm, Agilent technologies), thermostatted at 55 °C, at a flow rate of 0.05 ml/min. This column is suitable for peptide separation and analysis. After the injection, the columns were washed with buffer A for 5 min and the digested peptides were eluted using a linear gradient 0-40% B (buffer B: water/acetonitrile/formic acid, 10:89.9:0.1) for 90 min followed by a linear gradient 40-95% B for 20 min. The column was equilibrated in the initial conditions for 10 min before every injection.

The mass spectrometer was operated in the positive mode. The nebulizer gas pressure was set to 35 psi, whereas the drying gas flow was set to 14 l/min at a temperature of 300 °C, and the seath gas flow was set to 11 l/min at a temperature of 250 ºC. The capillary spray, nozzle, fragmentor and octopole RF Vpp voltages were 3500V, 100V, 360V and 750V respectively. Profile data were acquired for both MS and MS/MS scans in extended dynamic range mode. MS and MS/MS mass range was 50-1700 m/z and scan rates were 8 spectra/sec for MS and 3 spectra/sec for MS/MS. Auto MS/MS mode was used with precursor selection by abundance and a maximum of 20 precursors selected per cycle. A ramped collision energy was used with a slope of 3.6 and an offset of -4.8. The same ion was rejected after two consecutive scans.

Data processing and analysis was performed Spectrum Mill MS Proteomics Workbench (Rev B.06.00.201, Agilent Technologies, Santa Clara, CA, USA). Briefly, raw data were extracted under default conditions as follows: unmodified or carbamidomethylated cysteines; [MH]+ 50–10000 m/z; maximum precursor charge +5; minimum signal-to-noise MS (S/N) 25; finding ^{12}C signals.

The MS/MS search against the appropriate and updated protein database was performed with the following criteria: variable modifications search mode (carbamidomethylated cysteines, STY phosphorylation, oxidized methionine, and N-terminal glutamine conversion to pyroglutamic acid); tryptic digestion with 5 maximum missed cleavages; ESI-Q-TOF instrument; minimum matched peak intensity 50%; maximum ambiguous precursor charge +5; monoisotopic masses; peptide precursor mass tolerance 20 ppm; product ion mass tolerance 50 ppm; and calculation of reversed database scores. Validation of peptide and protein data was performed using auto thresholds.

Experimental parameters for HPLC and Q-TOF were set in MassHunter Workstation Data Acquisition software (Agilent Technologies, Rev. B.08.00) for free label quantification and identification (Kronstrand et al., 2018) in three pooled of concentrated supernatants from three different experiments and for each experimental line (WT PC; KO PC; WT PC + GB; KO PC + GB; control GB; control cell culture media). The proteins in the culture medium from negative controls (WT PC, KO PC, GB, control cell culture media) were subtracted from the KO PC + GB and WT PC + GB averages and the ratio of the averages was determined.

Immunofluorescence and microscopy: For detection of protein associated to the phagosome function (Supplementary Figure 2A), WT and KO PC, alone or cocultured with U87 and U373 GB lines, were 100% methanol-fixed, permeabilized with Triton X-100 0.1%, blocked with BSA and incubated with antibodies against mouse ATP6V1A (Abcam) and alpha-Smooth Muscle Actin (α-SMA; Abcam). Anti-Rabbit AlexaFluor 488 (Invitrogen) was used as secondary antibody against ATPase and Anti-Mouse Cyanine5 (Invitrogen) against α-SMA.

For *in vitro* phagocytosis assay (Supplementary Figure 3), a protocol similar to that described in Diaz-Aparicio et al. was followed (Diaz-Aparicio et al., 2016). Briefly, WT PC and KO PC were allowed to rest and settle for at least 48 h before phagocytosis experiments in 24-well plates. GB cell lines were previously labeled with the cell tracker DiI and treated with 60 μM of staurosporine (Cayman Chemical) for 48 h to induce apoptosis. Only the floating dead-cell fraction was collected from the supernatant and added to the PC cultures in a proportion of 1:1. Apoptotic cells were visualized and quantified by trypan blue exclusion. Because cell membrane integrity is still maintained in early induced apoptotic cells, cells not labeled with trypan blue were considered apoptotic. After 2 h, cells were fixed with 4% paraformaldehyde in PBS after washing away with media to discard all apoptotic cells non-trapped by PC. Due to its non-adherent nature, after cell washing, only apoptotic cells that have been trapped by PCs (in different stages of phagocytosis) remain. Remanent apoptotic cells trapped by PC and PC were stained with AlexaFluor 488-labeled Phalloidin (Invitrogen) to detect F-actin cytoskeleton.

Images were acquired with a Delta Vision RT (Applied Precision) restoration microscope coupled to a Coolsnap HQ camera (Photometrics), with a 60x/1.42 Plan Apo or 100x/1.40 Uplan Apo objectives. Morphometric measurements and quantification of cells, including histochemical quantification of phagocytic populations shown in Supplementary Figure 5, were performed using ImageJ (NIH, USA) software. Pictures for illustrations and quantitative analysis were uploaded from direct microscopic images and were not manipulated in subsequent steps of figures preparation, except for framing and scaling.

Flow cytometry analysis. Expression of MHC class I (H-2Kb, clone: AF6-88.5; BD Biosciences) and MHC class II (I-A/I-E, clone: M5/114.15.2; eBioscience,) were analyzed using DiI labeling solution (Invitrogen) for tracking and fluorescence separation of cells and specific anti-mouse antibodies. Background fluorescence was analyzed using labeled isotype monoclonal antibodies, and

GB cells were measured as negative controls to discard nonspecific labeling of human cells using anti-mouse antibodies. Stained cells were analyzed by flow cytometry using a FACSCanto flow cytometer (BD Biosciences) and data were analyzed with Flowjo analysis software (FlowJo, LLC).

For HCELL expression, wild type pericytes or LAMP-2A knock out pericytes were exofucosylated and analyzed for expression of sLeX with HECA452 antibody or control isotype by flow cytometry. All results are representative of at least four independent experiments using both wild type and LAMP-2A knock out pericytes.

References

Anders, S., Pyl, P.T., and Huber, W. (2015). HTSeq--a Python framework to work with high-throughput sequencing data. *Bioinformatics* 31(2)**,** 166-169. doi: 10.1093/bioinformatics/btu638.

Kim, D., Langmead, B., and Salzberg, S.L. (2015). HISAT: a fast spliced aligner with low memory requirements. *Nat Methods* 12(4)**,** 357-360. doi: 10.1038/nmeth.3317.

Kronstrand, R., Forsman, M., and Roman, M. (2018). Quantitative analysis of drugs in hair by UHPLC high resolution mass spectrometry. *Forensic Sci Int* 283**,** 9-15. doi: 10.1016/j.forsciint.2017.12.001.

Pascovici, D., Handler, D.C., Wu, J.X., and Haynes, P.A. (2016). Multiple testing corrections in quantitative proteomics: A useful but blunt tool. *Proteomics* 16(18)**,** 2448-2453. doi: 10.1002/pmic.201600044.

R Core Team (2021). R: A language and environment for statistical computing. *R Foundation for Statistical Computing, Vienna, Austria.*

Ritchie, M.E., Phipson, B., Wu, D., Hu, Y., Law, C.W., Shi, W., et al. (2015). limma powers differential expression analyses for RNA-sequencing and microarray studies. *Nucleic Acids Research* 43(7).

Sherry, S.T., Ward, M., and Sirotkin, K. (1999). dbSNP-database for single nucleotide polymorphisms and other classes of minor genetic variation. *Genome Res* 9(8)**,** 677-679.

2 Supplementary Figures and Table

(S1,A)

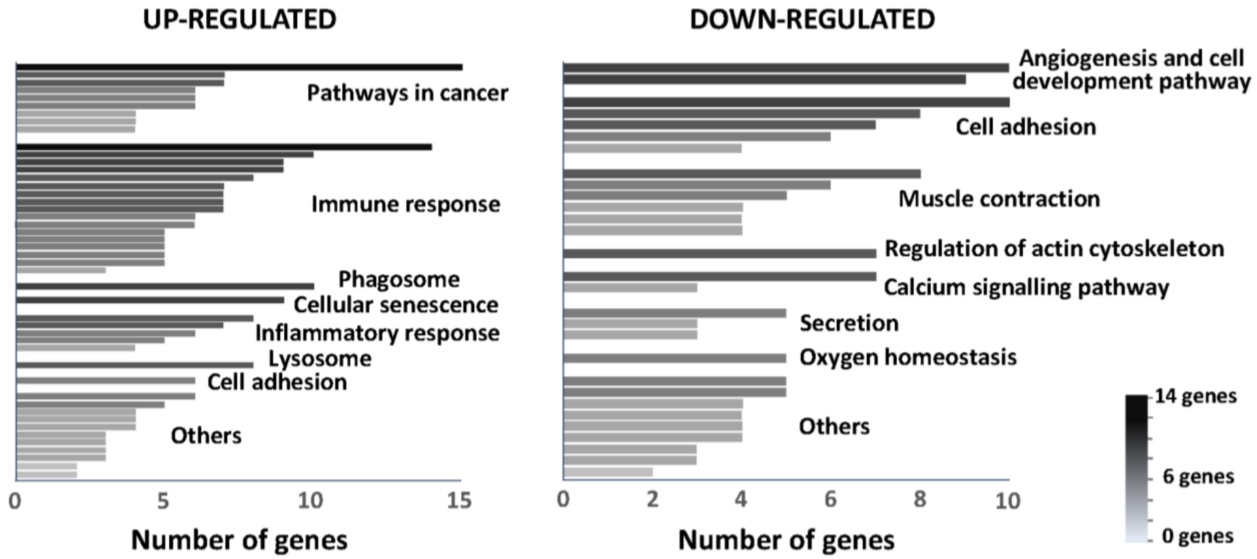

(S1,B)

UP-REGULATED							
id	symbol	description	Pathway 1	Pathway 2	Pathway 3	Pathway 4	Pathway 5
ENSMUSG00000073421	H2-Ab1	histocompatibility 2, class II antigen A, beta 1	X	X		X	X
ENSMUSG00000004371	Il11	interleukin 11				X	
ENSMUSG00000030162	Olr1	oxidized low density lipoprotein (lectin-like) receptor 1		X			
ENSMUSG00000028111	Ctsk	cathepsin K				X	
ENSMUSG00000020427	Igfbp3	insulin-like growth factor binding protein 3			X		
ENSMUSG00000073409	H2-Q6	histocompatibility 2 class I, Q region locus 6	X	X	X		X
ENSMUSG00000015533	Itga2	integrin alpha 2	X	X			
ENSMUSG00000029380	Cxcl1	chemokine (C-X-C motif) ligand 1				X	
ENSMUSG00000020695	Mrc2	mannose receptor, C type 2		X			
ENSMUSG00000023885	Thbs2	thrombospondin 2	X	X			
ENSMUSG00000073411	H2-D1	histocompatibility 2 class I, D region locus 1	X	X	X		X
ENSMUSG00000035929	H2-Q4	histocompatibility 2 class I, Q region locus 4	X	X	X		X
ENSMUSG00000052459	Atp6v1a	ATPase, H+ transporting, lysosomal V1 subunit A		X		X	
**DOWN-REGULATED**							
id	symbol	description	Pathway 1	Pathway 2	Pathway 3	Pathway 4	Pathway 5
ENSMUSG00000017344	Vtn	vitronectin		X			
ENSMUSG00000026678	Rgs5	regulator of G-protein signaling 5	X				
ENSMUSG00000042988	Notum	notum palmitoleoyl-protein carboxylesterase	X				
ENSMUSG00000005611	Mrvi1	MRV integration site 1			X		
ENSMUSG00000035305	Ror1	receptor tyrosine kinase-like orphan receptor 1	X				
ENSMUSG00000025321	Itgb8	integrin beta 8				X	
ENSMUSG00000026768	Itga8	integrin alpha 8				X	
ENSMUSG00000031616	Ednra	endothelin receptor type A			X		X
ENSMUSG00000025006	Sorbs1	sorbin and SH3 domain containing 1		X			
ENSMUSG00000026463	Atp2b4	ATPase, Ca++ transporting, plasma membrane 4					X
ENSMUSG00000051177	Plcb1	phospholipase C, beta 1	X		X		X
ENSMUSG00000025314	Ptprj	protein tyrosine phosphatase, receptor type, J		X			
ENSMUSG00000021823	Vcl	vinculin		X		X	
ENSMUSG00000022995	Enah	ENAH actin regulator				X	
ENSMUSG00000035783	Acta2	actin, alpha 2, smooth muscle, aorta			X		

Supplementary Figure 1.(**S1,A**) Kyoto Encyclopedia of Genes and Genomes (KEGG) pathway enrichment analysis corresponding to the network visualization shown in figure 1C. Biological pathways from CMA-dependent DEGs "up-regulated" or "down-regulated" in the KO PC compared to the WT PC in the presence of GB and the number of genes that they contain. The value of intensity is based on the number of genes. Data were obtained from 3 RNA pools for each experimental line, of at least, 5 independent experiments, using U87 GB line. (**S1,B**) Table showing the more up- or down-regulated genes in the main selected affected up- or down-regulated pathways from CMA-dependent DEGs, respectively, which are shown in figure 1C. The up-regulated pathways are the followings: Pathway 1: Antigen presentation and T-cell responses; Pathway 2: Phagosome; Pathway 3: Cellular Senescence; Pathway 4: Inflammatory response; Pathway 5: Cell adhesion (CAMs). The down-regulated pathways are the followings: Pathway 1: Angiogenesis and cell development pathway; Pathway 2: Cell adhesion (adherens junctions); Pathway 3: Vessel smooth muscle contraction; Pathway 4: Regulation of actin cytoskeleton; Pathway 5: Calcium signaling pathway. Data were obtained from 3 RNA pools for each experimental line, of at least, 5 independent experiments, using U87 GB cell line.

(S2,A)

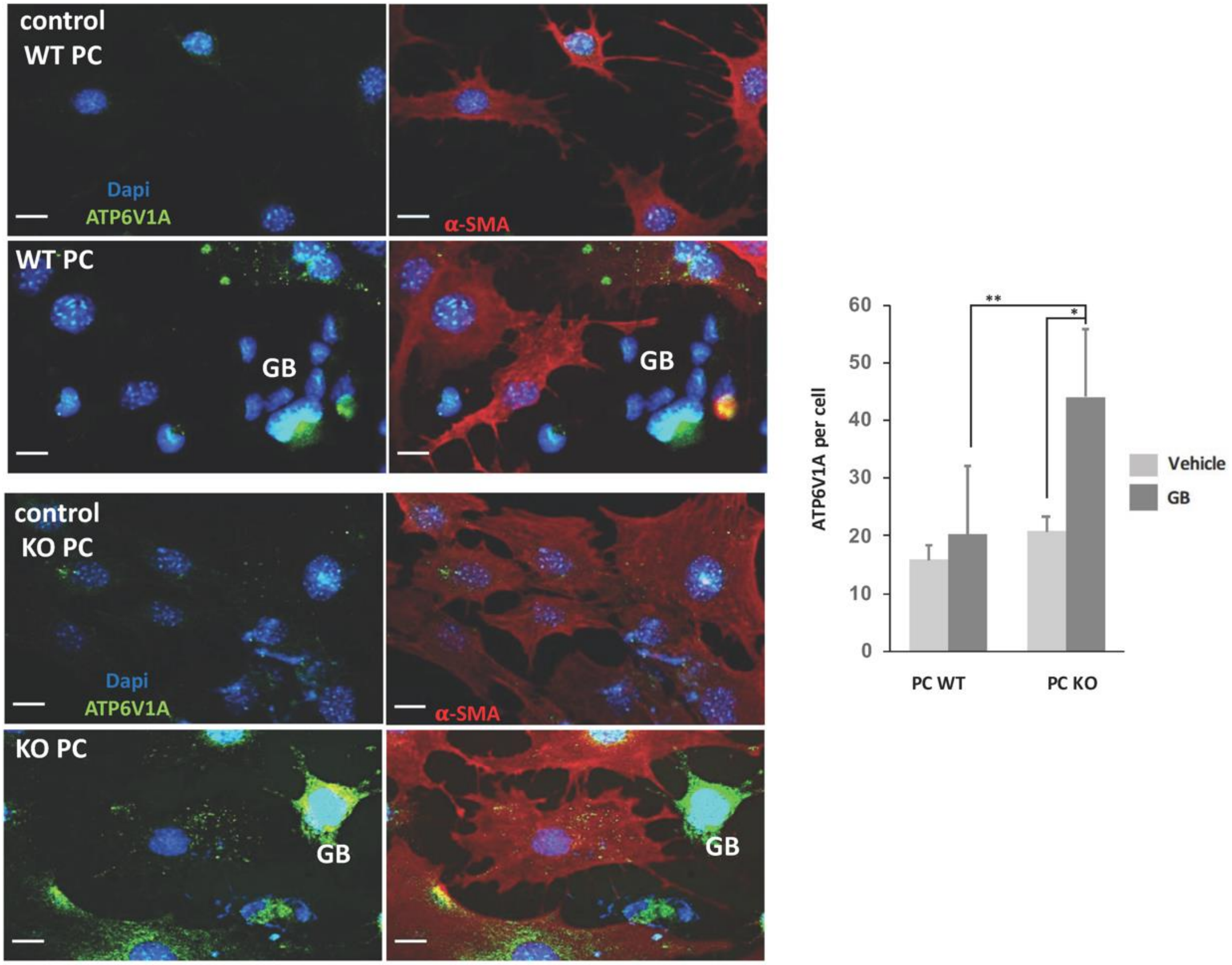
control
WT PC
Dapi
ATP6V1A
α-SMA
WT PC
GB
GB
control
KO PC
Dapi
ATP6V1A
α-SMA
KO PC
GB
GB
60
50
40
30
20
10
0
ATP6V1A per cell
**
*
Vehicle
GB
PC WT
PC KO

(S2,B)

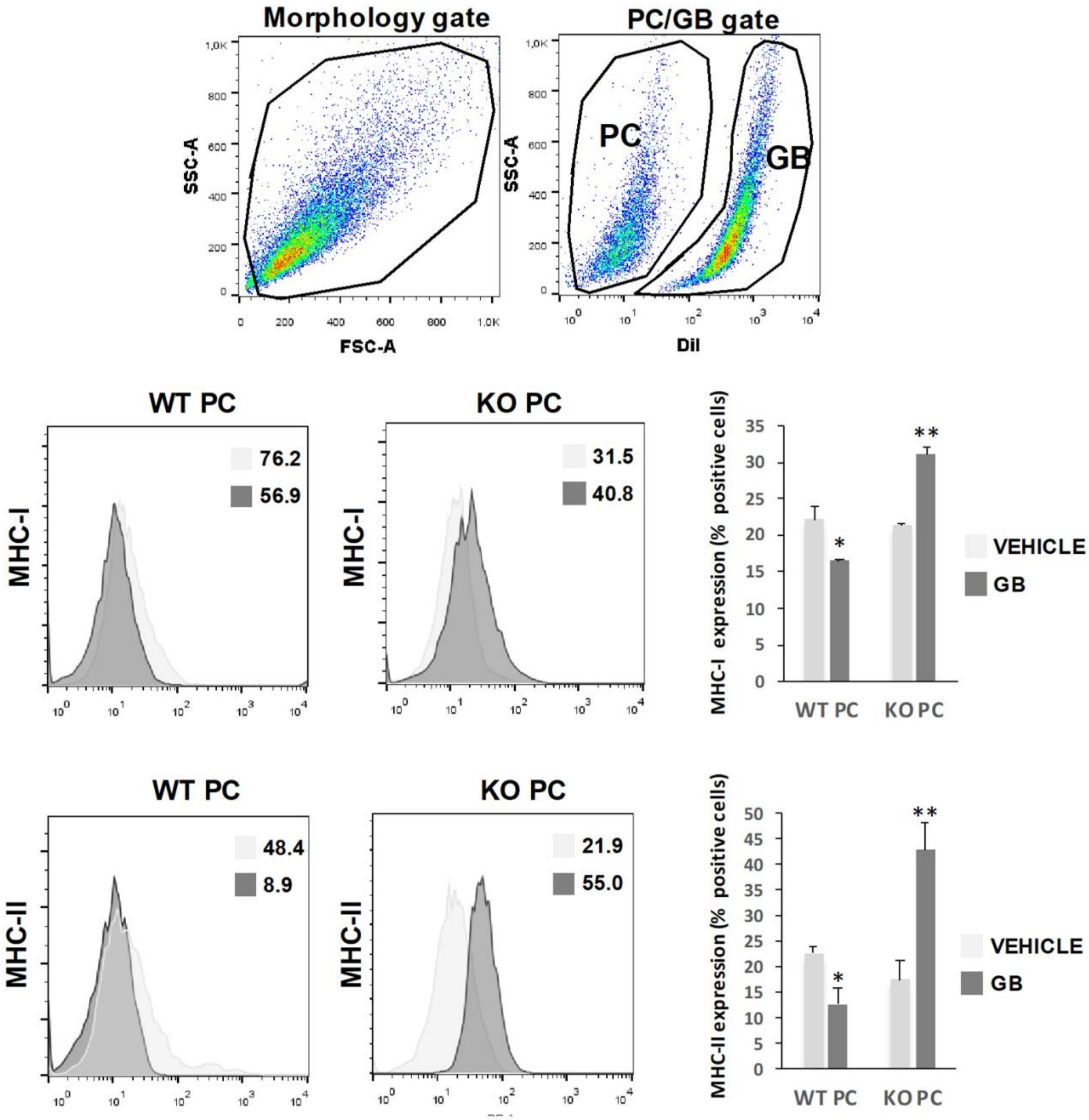
Morphology gate
PC/GB gate
SSC-A
FSC-A
PC
GB
DiI
WT PC
KO PC
MHC-I
76.2
56.9
31.5
40.8
MHC-I expression (% positive cells)
VEHICLE
GB
MHC-II
48.4
8.9
21.9
55.0
MHC-II expression (% positive cells)
VEHICLE
GB

Supplementary Figure 2. Protein expression of some validated gene and associated to the phagosome function. (**S2,A**) Expression of the catalytic subunit of the peripheral V1 complex of vacuolar ATPase (ATP6V1A; in green) in WT and KO PC, alone (control WT PC /KO PC) or cocultured with GB for 72 hours. Alpha-smooth muscle actin (α-SMA) was used to identify PC (in red). Nuclei of GB and PC were stained with DAPI (in blue). Scale bars: 50 μm. Shown images are using U87 GB line and are representative of at least, four experiments using both U87 and U373 GB lines. Graph shows the quantification of particles of ATP6V1A per cell from four independent experiments using U87 and U373 GB lines; * $P < 0.05$ and ** $P < 0.01$. (**S2,B**) Flow cytometry analysis of the expression of MHC-I and MHC-II molecules in WT and KO PC in absence (Vehicle) or presence of GB cells (GB) after 72 h of co-culture. Gating strategy to identify DiI positive GB-labeled cell population for further flow cytometry analyses is shown (upper panels). Insert numbers inside histograms represent mean fluorescence intensity (MFI) values. Bar graphs show percentages of expression of MHC-I and MHC-II in WT and KO PC in the different conditions. Nonspecific fluorescence was measured using specific isotype monoclonal antibodies, and GB cells were used as negative control (not shown). Data represents mean ± SD obtained from at least 5 independent experiments using U373 and U87 GB lines; *$P < 0.05$; **$P < 0.01$.

(S3)

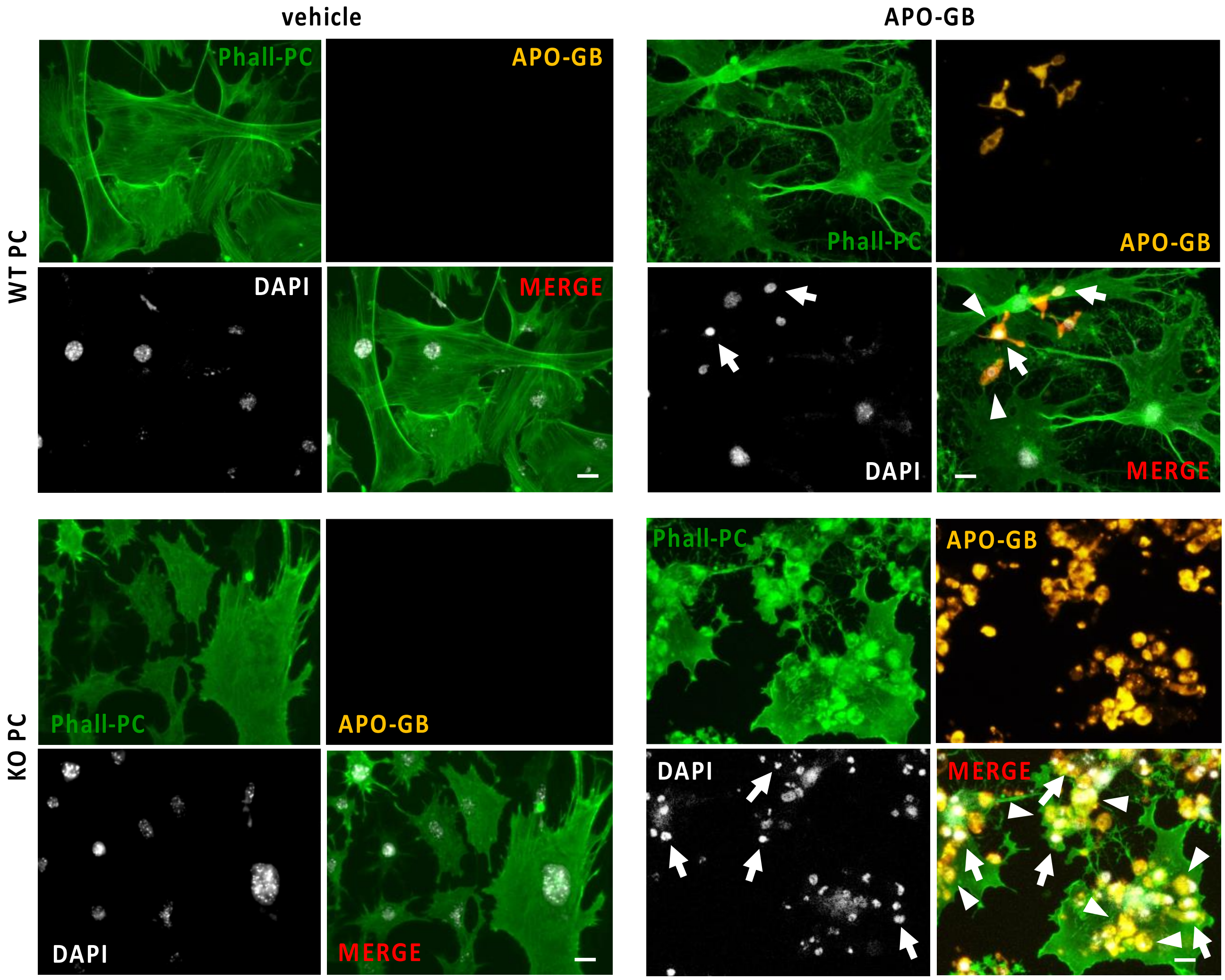

Supplementary Figure 3. Phagocytic capacity (Ph capacity) of KO PC (below) versus WT PC (above) against GB cells. Figure shows representative images of PC visualized with phalloidin (Phall-PC; green) displaying phagocytic activity by engulfing of pyknotic nuclei (with the DNA dye DAPI, white; arrows) and/or cytoplasmic inclusions (arrowheads) of apoptotic GB, stained with DiI and phalloidin (APO-GB; yellow), and compared to control PC without APO-GB (vehicle). Images are representative of five independent experiments of both GB cell lines (U373 and U87), using U87; Scale bars: 50 µm.

(S4)

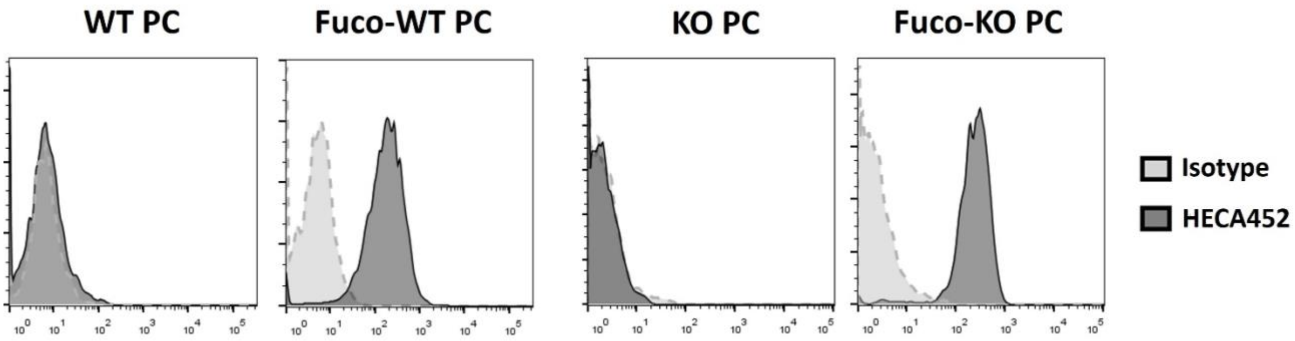

Supplementary Figure 4. Exofucosylation treatment enforced HCELL expression in pericytes. Wild type pericytes (WT PC, left) or LAMP-2A knock out pericytes (KO PC, right) were exofucosylated and analyzed for expression of sLeX with HECA452 antibody (dark grey histograms) or control isotype (dotted light grey histograms). PC only treated with GDP-fucose (WT PC or KO PC) did not stain with HECA452 antibody, whereas exofucosylation treatment with FTVII and GDP-fucose induced a robust sLeX expression in Fuco-WT PC or Fuco-KO PC, respectively. All results are representative of at least four independent experiments using both wild type and LAMP-2A knock out pericytes.

(S5,A)

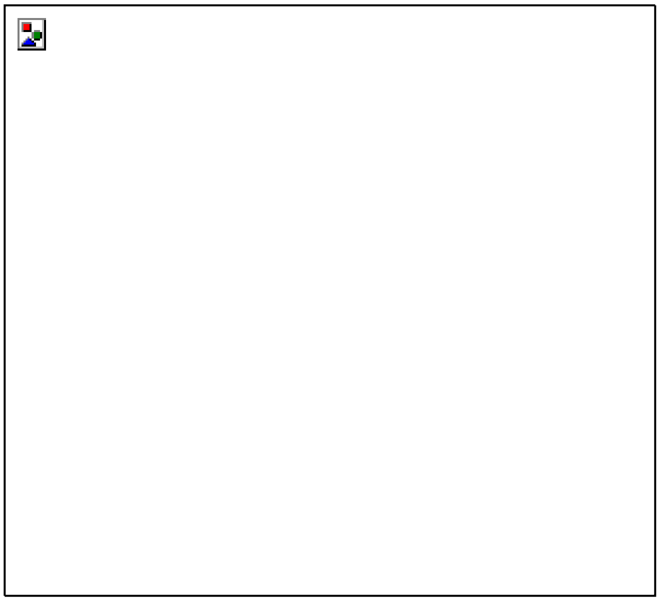

(S5,B)

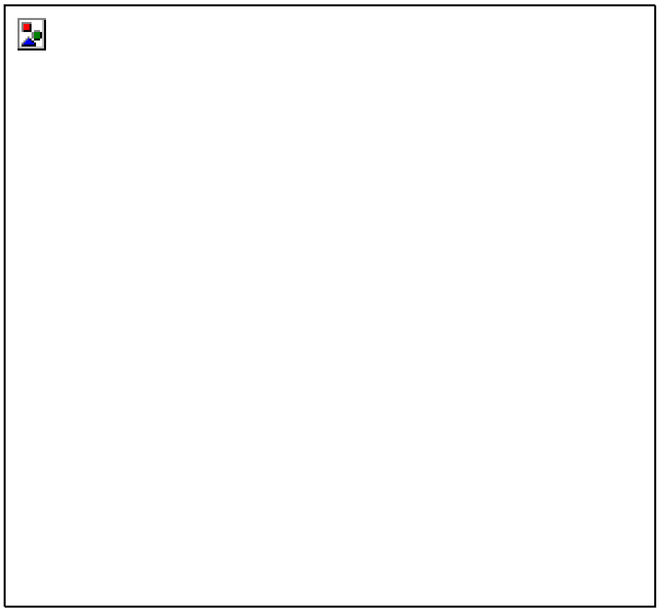

(S5,C)

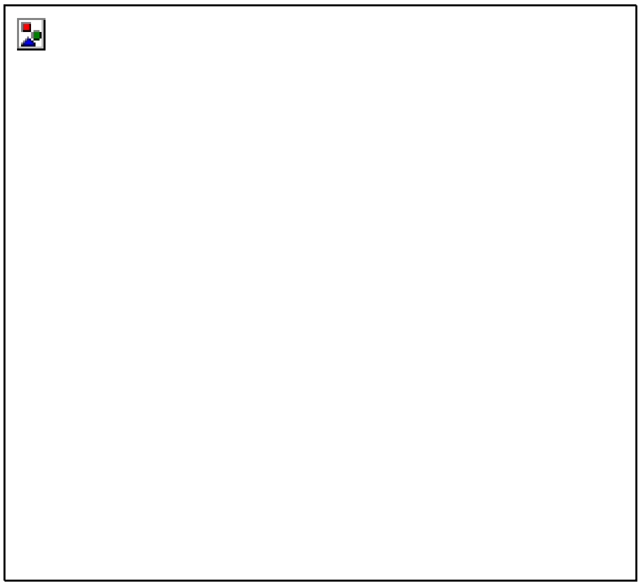

Supplementary Figure 5. Quantification of phagocytic populations. **(S5,A)** Quantification of the total number of Iba-1^{+} cells in each experimental line after therapy. **(S5,B)** Quantification of the number of perivascular cells immunopositive for Iba-1 staining, in each experimental line after therapy. **(S5,C)** Quantification of CD68 immunopositive cells in each experimental line. All graphs are showing mean ± SD obtained from at least, three independent experiments using both U373 and U87 GB lines; **p<0.01, ***p<0.005.
